# Structural basis for distinct protective mechanisms of IGHV3-23 antibodies targeting influenza hemagglutinin stem

**DOI:** 10.1038/s41467-026-74658-w

**Published:** 2026-06-22

**Authors:** Huibin Lv, Yang Wei Huan, Tossapol Pholcharee, Qi Wen Teo, Wenkan Liu, Akshita B. Gopal, Danbi Choi, Madison R. Ardagh, Timothy J. C. Tan, Yuanxin Sun, Arjun Mehta, Jinghang Li, Mateusz Szlembarski, Jessica J. Huang, Emily X. Ma, Lucas E. Wittenborn, Poorva Kasture, Chris K. P. Mok, Nicholas C. Wu

**Affiliations:** 1 Department of Biochemistry, University of Illinois Urbana-Champaign, Urbana, IL USA; 2 Carl R. Woese Institute for Genomic Biology, University of Illinois Urbana-Champaign, Urbana, IL USA; 3 Center for Biophysics and Quantitative Biology, University of Illinois Urbana-Champaign, Urbana, IL USA; 4 The Jockey Club School of Public Health and Primary Care, The Chinese University of Hong Kong, Hong Kong, China; 5 Li Ka Shing Institute of Health Sciences, Faculty of Medicine, The Chinese University of Hong Kong, Hong Kong, China; 6 S.H. Ho Research Centre for Infectious Diseases, The Chinese University of Hong Kong, Hong Kong, China; 7 School of Biomedical Sciences, The Chinese University of Hong Kong, Hong Kong, China; 8 Carle Illinois College of Medicine, University of Illinois Urbana-Champaign, Urbana, IL USA

**Keywords:** Antibodies, Influenza virus, Cryoelectron microscopy, Infection, Antimicrobial responses

## Abstract

Characterization of antibodies targeting the conserved stem domain of influenza hemagglutinin (HA) is critical for developing broadly protective countermeasures against the influenza virus. From a phage display human antibody library, this study discovers three group 1 HA-specific stem antibodies, namely HB31, HB34, and HB315, all of which are encoded by IGHV3-23. While HB31 and HB34 have minimal neutralization activity in vitro, their Fc-mediated effector functions lead to better in vivo protection than the potently neutralizing HB315. Consistently, cryo-EM analysis suggests that HB31 and HB34 have a higher Fc accessibility than HB315, based on their epitopes and approaching angles. HB31 and HB34 engage a pocket in the upper HA stem that is rarely targeted by known HA stem antibodies, whereas the epitope of HB315 involves the lower stem. Overall, our findings provide insights not only into the structure-function relationship of HA stem antibodies but also into the design of next-generation influenza therapeutics.

## Abstract (web summary)

Antibodies that target the stem region of the influenza haemagglutinin are sought because this region is conserved between different clades and subtypes of the virus. Here, the authors characterize three stem reactive human antibodies and show functional neutralization and binding specificity, showing differences in binding site as well as Fc-dependent effector function.

## Introduction

Influenza remains a persistent global health threat, causing an estimated 250,000 to 500,000 deaths annually^1^. Currently, H1N1 and H3N2 subtypes of influenza A viruses, as well as Victoria lineage of influenza B viruses, are circulating in human^2,3^. Influenza A viruses have caused at least four major pandemics in the past, including the 1918 H1N1 “Spanish flu”, the 1957 H2N2 “Asian flu”, the 1968 H3N2 “Hong Kong flu”, and the 2009 H1N1 “swine flu”^4^. In addition, highly pathogenic avian influenza H5 and H7 subtypes are posing serious pandemic threats, with case fatality rates up to 60%^5^. In 2024, an outbreak of avian influenza H5N1 virus clade 2.3.4.4b occurred in Texas dairy cattle^6^. Since then, more than 1,000 dairy herds in the US have been infected by avian influenza H5N1 virus^7^, along with at least 70 confirmed human cases, including two fatalities^8^.

Influenza A viruses encode two major surface glycoproteins, hemagglutinin (HA) and neuraminidase (NA). HA is the most abundant and immunodominant surface antigen, serving as the primary target of neutralizing antibodies elicited by infection or vaccination^9^. To date, 19 HA subtypes (H1-H19) have been identified, which are phylogenetically categorized into group 1 (H1, H2, H5, H6, H8, H9, H11, H12, H13, H16, H17, H18, H19) and group 2 (H3, H4, H7, H10, H14, H15)^10^. HA is expressed as a single-chain precursor (HA0) and assembles into a homotrimer. HA0 is then cleaved by host proteases into disulfide-linked HA1 and HA2 subunits^10^. HA contains an immunodominant head domain, which is composed entirely of HA1, resides atop the membrane-proximal stem domain, which is primarily formed by HA2. The HA head domain binds to the host sialylated receptors and is highly variable, whereas the stem domain harbors the virus-host membrane fusion machinery and is more conserved across strains and subtypes^11^. As a result, antibodies targeting the immunosubdominant HA stem often exhibit broad neutralizing activity against antigenically diverse strains and subtypes^10^.

Over the past two decades, many HA stem antibodies with broad cross-reactivity and protection activity have been identified^12–18^. Analysis of the HA stem antibody repertoires has uncovered several recurring sequence features, mainly defined by immunoglobulin heavy variable (*IGHV*) gene usage and sequence motifs within the complementarity-determining region (CDR) H3. Notable examples include *IGHV6-1* with an *IGHD3-3*-encoded CDR H3 FG[I/V/L] motif^13,19,20^, *VH1-18* with a QxxV motif^19,21^, and *IGHD3-9*-encoded LxYFxWL motif^22,23^. Two major neutralizing epitopes were discovered for the group 1 HA stem, namely the central stem epitope and the anchor epitope^24–28^. While central stem antibodies often broadly recognize multiple subtypes^24–26^, anchor antibodies are usually more subtype-restricted^27,28^. Structural studies have revealed that central stem antibodies frequently target five hydrophobic pockets within the stem domain using aromatic and hydrophobic side chains^10,29^. The discovery and characterization of HA stem antibodies have motivated the development of broadly protective influenza vaccines^30–32^, some of which have shown promising results in phase I clinical trials^33–36^. HA stem antibodies have also guided structure-based design of proteins^37–41^, peptides^29,42^, and small molecules^43–45^ with neutralization activity.

In this work, we identify three *IGHV3-23* HA stem antibodies, HB31, HB34, and HB315, from a phage display human antibody library that was previously constructed from 245 healthy donors^46^. All three antibodies bind to HAs from multiple group 1 subtypes, including one from the H5N1 clade 2.3.4.4b. HB31 and HB34 primarily rely on Fc-mediated effector functions for their in vivo protection activity, whereas HB315 does not. Such a difference can be attributed to their epitopes and the approach angles revealed by cryo-EM analysis. Throughout this study, HA residue positions are numbered according to the H3 HA reference, and antibody residues are numbered using Kabat nomenclature.

## Results

### Discovery of HA stem antibodies by phage display and long-read sequencing

**Fig. 1:**
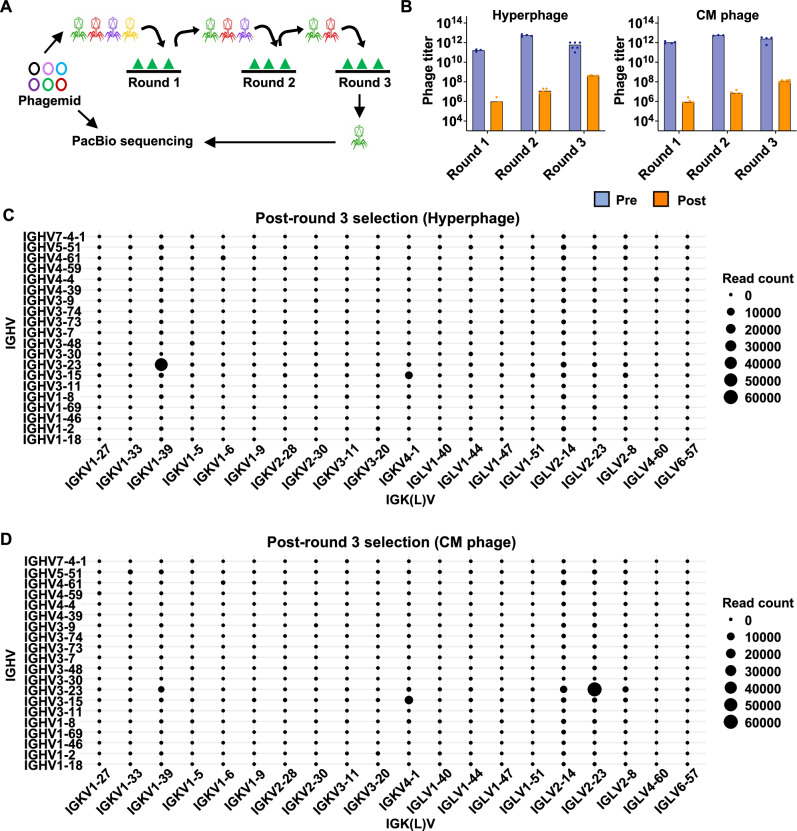
Antibody screening against the HA stem by phage display. **A** Three rounds of panning were performed against the HA stem, followed by PacBio sequencing of both the phagemid antibody library before panning and after three rounds of panning. **B** Phage titers were measured before (Pre, blue) and after (Post, orange) each round of panning. Distribution of *IGHV/IGK(L)V* pairings following three rounds of panning of **C** Hyperphage antibody library or **D** CM13 phage antibody library. Point size reflects the sequencing read count for each *IGHV/IGK(L)V* combination.

We previously constructed a phagemid antibody library using the peripheral blood mononuclear cells (PBMCs) from 245 healthy individuals^46^. Here, this antibody library was displayed on M13 KO7 ΔpIII hyperphage (hyperphage) and CM13 interference-resistant helper phage (CM phage) (Fig. 1A). These two phage display antibody libraries were subjected to three rounds of panning against an HA stem construct derived from the A/Brisbane/59/2007 (H1N1) HA (hereafter referred to as the H1 stem)^32^ (Fig. 1A). For both phage display antibody libraries, the titer increased from round to round, indicating enrichment of phage clones encoding antibodies that bound to the H1 stem (Fig. 1B). The occurrence frequencies of individual clones in the input phagemid antibody library and post-round three selection phage display antibody libraries was analyzed by PacBio long-read sequencing.

**Fig. 2:**
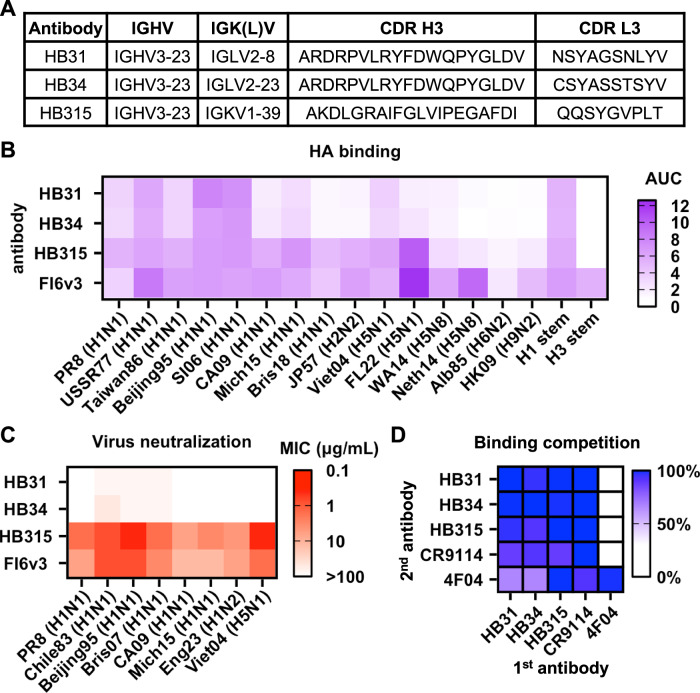
Binding and neutralization activity of HB31, HB34, and HB315. **A** Sequence information of three HA stem antibodies. **B** Binding activity of the indicated antibodies in IgG format to various HA proteins was assessed by ELISA and quantified as the area under the curve (AUC). **C** Neutralization activity of three HA stem antibodies was determined by microneutralization assay. FI6v3, which is a human HA stem antibody^14^, served as a positive control. MIC: minimum inhibitory concentration. **B**, **C** Strain names are abbreviated as follows: PR8: A/Puerto Rico/8/1934 (H1N1), USSR77: A/USSR/90/1977 (H1N1), Chile83: A/Chile/1/1983 (H1N1), Taiwan86: A/Taiwan/01/1986 (H1N1), Beijing95: A/Beijing/262/1995 (H1N1), SI06: A/Solomon Island/3/2006 (H1N1), Bris07: A/Brisbane/59/2007 (H1N1), CA09: A/California/07/09 (H1N1), Mich15: A/Michigan/45/2015 (H1N1), Bris18: A/Brisbane/02/2018 (H1N1), Eng23: A/England/234600203/2023 (H1N2), JP57: A/Japan/305/1957 (H2N2), Viet04: A/Vietnam/1203/2004 (H5N1), FL22: A/bald eagle/Florida/W22–134-OP/2022 (H5N1), WA14: A/northern pintail/WA/40964/2014 (H5N8), Neth14: A/chicken/Netherlands/14015531/2014 (H5N8), Alb85: A/mallard/Alberta/566/1985 (H6N2), and HK09: A/Hong Kong/33982/2009 (H9N2). **D** Competition among the indicated antibodies for binding to HA H1N1 A/Michigan/45/2015 (H1N1) was measured by biolayer interferometry (BLI). All antibodies were in Fab format. The percentage of binding competition is shown as a heatmap.

While the occurrence frequencies of different *IGHV/IGK(L)V* gene pairs were quite evenly distributed in the input phagemid antibody library (Fig. S1), certain *IGHV/IGK(L)V* gene pairs became enriched post-selection (Fig. 1C, D). After three rounds of selection, *IGHV3-23/IGLV2-23* and *IGHV3-23/IGKV1-39* had the highest occurrence frequency in the CM phage and hyperphage libraries, respectively. Our downstream analyses focused on two representative antibodies from these *V* gene families, namely HB34 (*IGHV3-23/IGLV2-23*) from the CM phage library and HB315 (*IGHV3-23/IGKV1-39*) from the hyperphage library (Figs. 2A, S2). We also included another antibody, HB31 (*IGHV3-23/IGLV2-8*), which had the same heavy chain variable domain sequence as HB34 but a different light chain (Figs. 2A, S2). HB31, HB34, and HB315 all reached high occurrence frequencies after three rounds of selection and were validated by ELISA to bind H1 stem (Fig. 2B).

### HB31, HB34, and HB315 are group 1 HA-specific antibodies

To evaluate the cross-reactivity breadth of HB31, HB34, and HB315, we assessed their binding activity against a panel of HAs from different subtypes. The three antibodies bound to all tested HAs from human H1N1 strains isolated between 1977 and 2018. Nevertheless, enzyme-linked immunosorbent assay (ELISA) showed that HB31 and HB34 exhibited 2- to 4-fold weaker binding activity than HB315 against HAs from post-2009 pandemic H1N1 strains, including A/California/04/2009 (H1N1), A/Michigan/45/2015 (H1N1), and A/Brisbane/02/2018 (H1N1) (Fig. 2B). Similarly, the binding activity of HB31 and HB34 to HAs from H2, H5, H6, and H9 subtypes was at least 2-fold weaker than HB315 (Fig. 2B). Despite the better binding activity and breadth of HB315, its binding activity to HAs from certain H5 strains, including A/northern pintail/WA/40964/2014 (H5N8), A/chicken/Netherlands/14015531/2014 (H5N8), as well as HAs from the H6 and H9 subtype was at least 2-fold weaker than the prototypic cross-group HA stem antibody FI6v3^14^ (Fig. 2B). Additionally, none of the three antibodies bound to a stabilized stem construct derived from H3N2 A/Finland/486/2004 HA (group 2 HA)^47^, indicating that their binding activity was specific to group 1 HA (Fig. 2B).

We further tested the neutralizing activity of HB31, HB34, and HB315 against a panel of 7:1 reassortants carrying HAs from human H1N1 strains, avian A/Vietnam/1203/2004 (H5N1), and swine A/England/234600203/2023 (H1N2) in A/Puerto Rico/8/1934 (PR8, H1N1) backbone. Among the three antibodies, HB315 exhibited the most potent neutralization activity across all tested viruses (Fig. 2C). The neutralization potency of HB315 was comparable to, or up to 4-fold greater than, that of FI6v3 against the tested viruses (Fig. 2C). Conversely, HB31 and HB34 were at least 50-fold less potent than HB315 against A/Chile/1/83 (H1N1), A/Beijing/262/95 (H1N1), and A/Brisbane/59/2007 (H1N1), and exhibited no detectable activity against the remaining strains, even at 100 μg/mL (Fig. 2C). Collectively, HB315 demonstrated superior neutralization potency and breadth compared to HB31 and HB34.

### HB31, HB34, and HB315 bind to central stem epitope

To probe the epitopes of HB31, HB34, and HB315, we performed a binding competition assay using biolayer interferometry. This binding competition assay also included 047-09 4F04 (known as 4F04 hereinafter)^27,28^ and CR9114^12^ as controls, which are prototypic antibodies to the anchor and central stem epitopes, respectively. HB31, HB34, and HB315 showed 90%-100% competition with CR9114 (Figs. 2D, S3). By contrast, they exhibited no competition with 4F04 when 4F04 was added first (Figs. 2D, S3). These results suggested that HB31, HB34, and HB315 targeted the central stem epitope, similar to CR9114. Although HB31, HB34, and HB315 competed strongly with 4F04 when added first (Figs. 2D, S3), this observation was likely due to steric hindrance blocking access to the 4F04 epitope, which is located closer to the biosensor surface. Consistently, CR9114 had no competition with 4F04 when 4F04 was added first, but showed strong competition when the order was reversed.

### HB31, HB34, and HB315 confer in vivo protection via different mechanisms

**Fig. 3:**
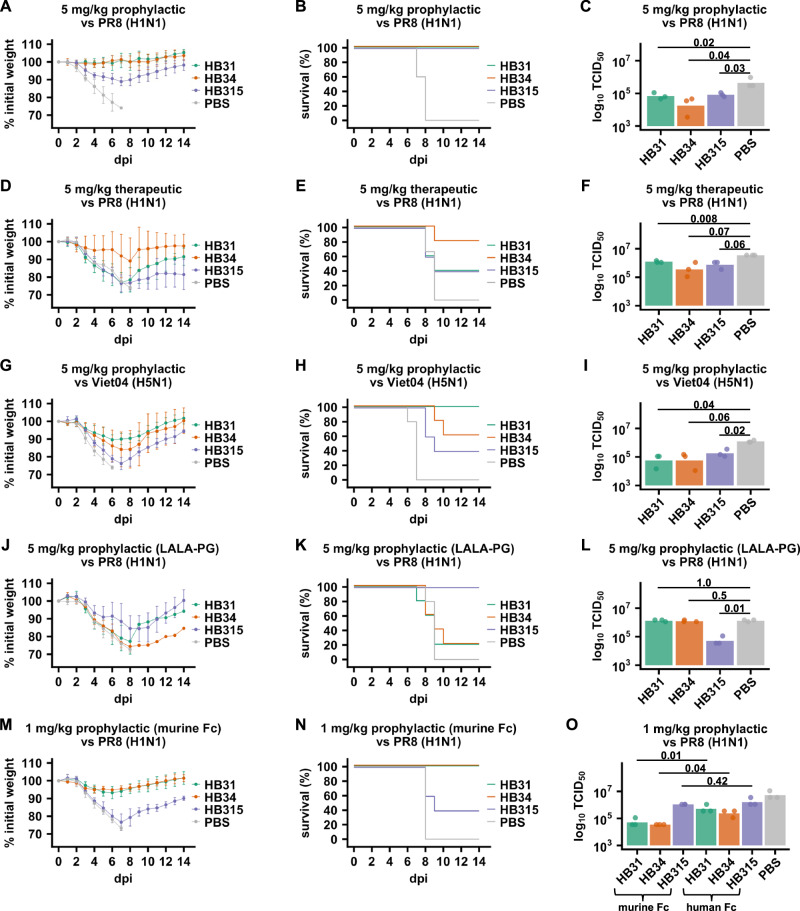
In vivo protection activity of HB31, HB34, and HB315. Female BALB/c mice at 6 weeks old were injected intraperitoneally with the indicated antibodies at the indicated dose (**A**–**C**, **G**–**O**) 4 hours prior to or (**D**–**F**) 24 hours after challenge with 5× LD_50_ of the indicated virus. PR8: A/Puerto Rico/8/1934 (H1N1). Viet04: recombinant PR8 virus carrying the HA from A/Vietnam/1203/2004 (H5N1). A total of 8 mice were used per experimental group (*n* = 5 for survival and weight monitoring; *n* = 3 for measuring lung viral titers). **A**, **D**, **G**, **J**, **M** The mean percentage of body weight change post-infection is shown (*n* = 5 per group). The error bars represent the SD. The humane endpoint was defined as a weight loss of 25% from initial weight on day 0. **B**, **E**, **H**, **K**, **N** Kaplan-Meier survival curves are shown (*n* = 5 per group). **C**, **F**, **I**, **L**, **O** Lung viral titers on day 3 post-infection are shown (*n* = 3 per group). *P*-values, which were computed by a two-tailed t-test, were indicated. Of note, while our BALB/c mice were not modified to facilitate interaction with human IgG1, human IgG1 could interact with murine Fc gamma receptors^87,88^.

To test the in vivo protection activity of HB31, HB34, and HB315, all three antibodies were administered prophylactically prior to a lethal challenge with the PR8 virus. According to the weight loss profiles (Fig. 3A), survival analysis (Fig. 3B), and lung viral titer at day 3 post-infection (Fig. 3C), all three antibodies at 5 mg/kg conferred protection against PR8. Nevertheless, the protection activity of HB31 and HB34 was stronger than HB315. With 1 mg/kg of antibodies, the survival rates of mice treated with HB31, HB34, and HB315 were 80% (4/5), 80% (4/5), and 20% (1/5), respectively (Fig. S4A, B). At 0.3 mg/kg, none of the mice treated with HB315 survived, whereas the survival rates of the mice treated with HB31 and HB34 were 60% (3/5) and 40% (2/5), respectively (Fig. S4C, D). Moreover, therapeutic administration of the three antibodies at 5 mg/kg conferred partial protection against PR8 infection, with survival rates of 60% (3/5), 80% (4/5), and 60% (3/5) in mice treated with HB31, HB34, and HB315, respectively (Fig. 3D–F). Furthermore, we tested the prophylactic protection activity of these antibodies at 5 mg/kg against a lethal challenge with A/Vietnam/1203/2004 (H5N1). While all three antibodies reduced the lung viral titer on day 3 post-infection (*p* = 0.02 to 0.06, two-tailed t-test) (Fig. 3I), only HB31 conferred complete protection, resulting in 100% survival rate of H5N1-infected mice (Fig. 3G, H). On the other hand, HB34 and HB315 conferred only partial protection, with survival rates of 60% (3/5) and 40% (2/5), respectively (Fig. 3G, H). Together, these results revealed an inverse relationship between neutralization potency and protection activity, with HB31 and HB34 exhibiting the weaker neutralization potency in vitro but conferring the stronger protection activity in vivo than HB315.

Previous studies have shown that some HA stem antibodies confer protection in vivo primarily through Fc-mediated effector functions^48–50^. To test if Fc-mediated effect functions of HB31, HB34, and HB315 contributed to their protective activity in vivo, we generated a LALA-PG variant for each of the three antibodies to eliminate their Fc effector functions^51^ (Fig. S5A, B). Notably, the binding and neutralization activities of the LALA-PG variants were comparable to their parental antibodies (Fig. S5C, D). While all mice treated with 5 mg/kg of HB315-LALA-PG were protected against PR8, the survival rate of mice treated with 5 mg/kg of HB31-LALA-PG or HB34-LALA-PG was only at 20% (1/5) (Fig. 3J, K). Additionally, mice treated with HB31-LALA-PG or HB34-LALA-PG had similar lung viral titer at day 3 post-infection as those without antibody treatment (*p* = 1.0 and 0.5, respectively, two-tailed t-test) (Fig. 3L). In comparison, the lung viral titer at day 3 post-infection in mice treated with HB315-LALA-PG was reduced by more than 10-fold (*p* = 0.01, two-tailed t-test) (Fig. 3L). These results suggested that Fc effector functions were more important for the in vivo protection activity of HB31 and HB34 than for that of HB315. Furthermore, HB31 and HB34 with murine Fc conferred complete protection against PR8 at 1 mg/kg (Fig. 3M, N). By contrast, HB315 with murine Fc only partially protected against PR8, with a survival rate of 40% (2/5) (Fig. 3M, N). HB31 and HB34 with murine Fc also reduced lung viral titers by approximately 10-fold compared to their human Fc counterparts (*p* = 0.01 and 0.04, respectively, two-tailed t-test), whereas no such difference was observed for HB315 (*p* = 0.42, two-tailed t-test) (Fig. 3O**)**. Consistently, HB31 and HB34 elicited stronger antibody-dependent cell-mediated cytotoxicity (ADCC) activity than HB315 in an in vitro reporter assay **(**Fig. S5A, B**)**.

### The epitope of HB315 overlaps with but is distinct from that of HB31 and HB34

**Fig. 4:**
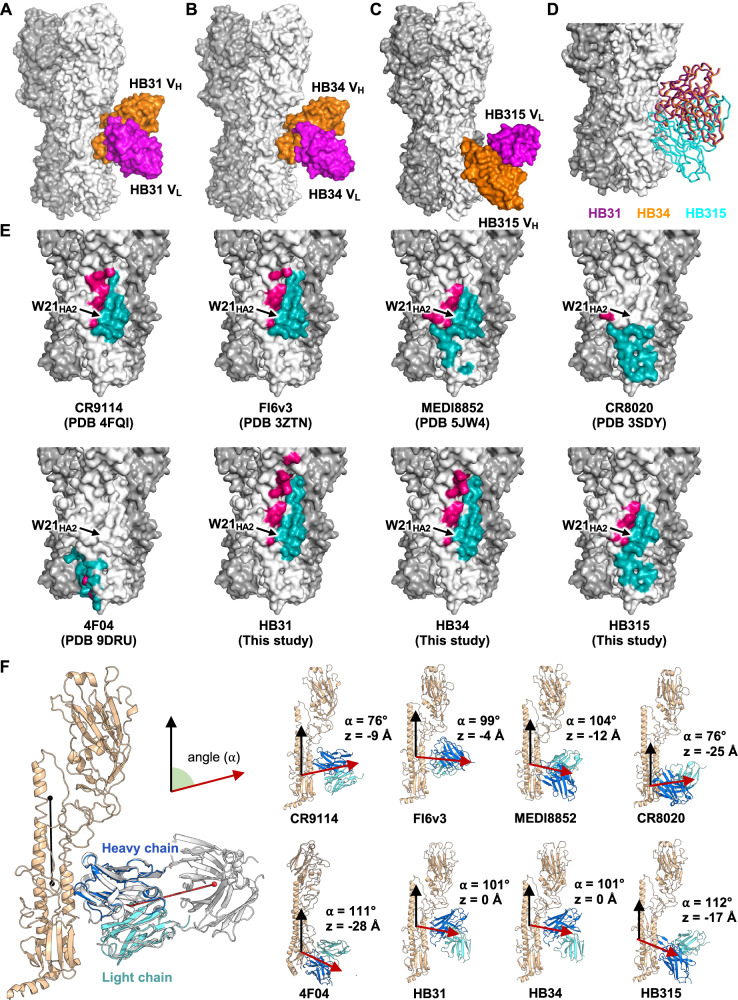
Epitopes and approaching angles of HB31, HB34, and HB315. **A**–**C** Cryo-EM structures of the indicated antibodies in complex with A/Solomon Island/3/06 (H1N1) HA. Antibodies and HA are shown in surface representation. HA is in white. Heavy chain variable domain (V_H_) and light chain variable domain (V_L_) are in orange and magenta, respectively. **D** The structures of HB31 (magenta), HB34 (orange), and HB315 (blue) in complex with HA are aligned based on HA. **E** Epitopes of the indicated antibodies are shown on the HA surface. HA1 and HA2 contacts are colored in pink and blue, respectively. The location of the highly conserved W21_HA2_ is indicated. **F** Fab binding angle (α) and z-coordinate (z) of the center of mass of each variable domain is quantified (see **Methods**). Black and red arrows represent the vectors along the HA stem and through the centers of mass of the variable domain, respectively. The HA and Fab are shown as cartoons (wheat: HA, blue: heavy chain, and cyan: light chain). **E**, **F** CR9114: PDB 4FQI^12^, FI6v3: PDB 3ZTN^14^, MEDI8852: PDB 5JW4^13^, 4F04: PDB 9DRU^28^, and CR8020: PDB 3SDY^17^.

To understand the biophysical basis of the distinct protection mechanisms among HB31, HB34, and HB315, we determined the cryo-EM structure of the fragment antigen-binding (Fab) of HB31, HB34, and HB315 in complex with A/Solomon Islands/3/2006 (H1N1) HA to a resolution of 2.76 Å, 2.64 Å, and 2.67 Å, respectively (Figs. 4A–C, S6, S7, S8, and Table S1). Consistent with the competition assay results (Figs. 2D, S3), our structural analysis showed that all three antibodies interacted with the central stem epitope (Fig. 4A–E), with paratopes composed exclusively of heavy chains (Fig. S9A–C). Since HB31 and HB34 shared the same heavy chain sequence, their binding modes were almost identical. Nevertheless, their light chain sequences differed, resulting in distinct heavy-light chain interfaces. A notable difference was the tighter packing of V_L_ Y96 with the heavy chain in HB31 compared to HB34 (Fig. S9D). In HB31, V_L_ Y96 H-bonded with V_H_ Y100g and was 7 Å from V_H_ L100i. In HB34, V_L_ Y96 H-bonded with V_H_ R96 and was only 3 Å from V_H_ L100i (Fig. S9D). This difference is likely responsible for the higher thermostability of HB34 (T_m_ = 68 °C) relative to HB31 (T_m_ = 66.5 °C) (Fig. S9E). HB31 and HB34 bound at least 4 Å higher on the HA stem than canonical central stem antibodies, such as CR9114^12^, FI6v3^14^, and MEDI8852^13^ (Fig. 4F). In contrast, HB315 bound lower than canonical central stem antibodies. Compared to HB31 and HB34, HB315 bound 17 Å lower on the HA stem, yet remained above the lower stem antibody CR8020 and the anchor antibody 4 F04. HB315 also exhibits a more downward binding angle (112°) than HB31 and HB34 (101°) (Fig. 4F). This approaching angle and binding lower on the HA stem likely limited the Fc-accessibility of HB315 to effector cells, which could help explain its lower ADCC activity compared to that of HB31 and HB34 (Fig. S5A, B), as well as its minimal reliance on Fc-mediated effector functions for protection activity in vivo (Fig. 3J–L).

The epitope of HB315 closely resembled that of the cross-group neutralizing antibody MEDI8852^13^ (Fig. 4E). HB315 had an *IGHD3-3*-encoded FGL motif at the tip of CDR H3 that was chemically similar to the YGV motif of MEDI8852 at equivalent positions (Fig. S10A–C). However, HB315 engaged the lower HA stem more extensively compared to MEDI8852 (Fig. 4E), mainly due to the H-bonds between its CDR H2 and the side chains of D19_HA2_, S32_HA2_, Y34_HA2_, and K153_HA2_ (Fig. S10A, B). Our binding data above showed that the binding activity of HB315 to H2N2 HA was at least 4-fold stronger than HB31 and HB34 (Fig. 2B). This difference was likely due to how they engaged HA2 residue 45 (Fig. S10D–G). Although most HA subtypes possess an Ile at HA2 residue 45, human H2N2 HA has a Phe at this residue, which is known to limit the cross-reactivity of HA stem antibodies^25,52^. HB31, HB34, and HB315 all interacted with I45_HA2_ via their CDR H3. At the same time, the CDR H3s of HB31 and HB34, but not HB315, were stabilized by light chain interactions (Fig. S10G). Therefore, the bulkier Phe45 residue in H2N2 HA could be accommodated by the less constrained CDR H3 of HB315, whereas the more rigid CDR H3s of HB31 and HB34 would result in steric hindrance. This structural analysis explained why HB315, but not HB31 or HB34, strongly cross-reacted with H2N2 HA.

### HB31 and HB34 extend binding to an additional pocket

**Fig. 5:**
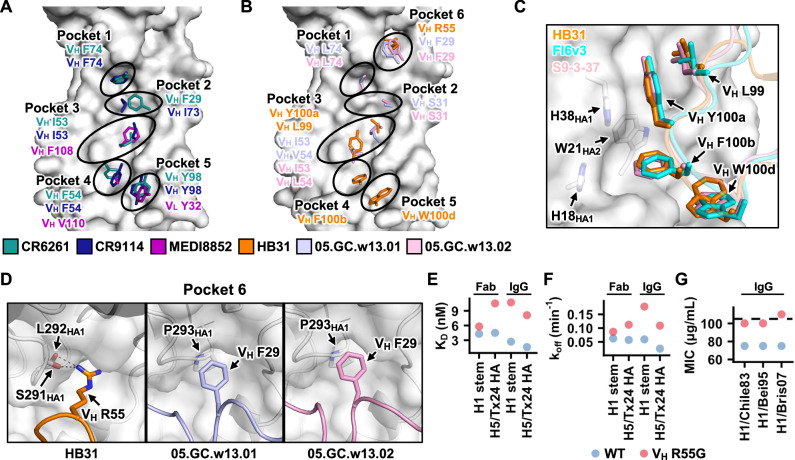
Structural features of HB31. **A** Five pockets in the HA stem that are targeted by known central stem antibodies are indicated on HA (white surface)^24^. Side chains of paratope residues in human HA stem antibodies CR6261 (PDB 3GBM)^24^, CR9114 (PDB 4FQI)^12^, and MEDI8852 (PDB 5JW4)^13^ that interact with these five pockets are shown as sticks. **B** Side chains of paratope residues in human HA stem antibodies HB31, 05.GC.w13.01 (PDB 8TXP)^53^ and 05.GC.w13.02 (PDB 8TXM)^53^ that interact with pockets 1-6 in the HA stem are shown as sticks. **C** Interactions between HA stem and the *IGHD3-9*-encoded LxYFxW motifs in HB31, FI6v3 (PDB 3ZTN)^14^, and S9-3-37 (PDB 6E3H)^22^ are shown. Their CDR H3s are shown as cartoon representations with the side chains of the LxYFxW motifs as sticks. HA is shown as a white semitransparent surface with the side chains of key epitope residues as sticks. **D** Interactions of pocket 6 with HB31, 05.GC.w13.01 (PDB 8TXP)^53^ and 05.GC.w13.02 (PDB 8TXM)^53^ are compared. CDR H2s are shown as cartoon representation. Key interacting residues are shown as sticks. **E** The binding affinity (K_D_) and **F** dissociation rate (k_off_) for HB31 wild type (WT, blue) or V_H_ R55G revertant (red) in Fab or IgG format against H1 stem or A/Texas/37/2024 (H5N1) HA are shown. **G** Neutralization activity of HB31 wild type (WT, blue) or V_H_ R55G revertant (red) in IgG format against the indicated H1N1 strains. Chile83: A/Chile/1/83 (H1N1), Bei95: A/Beijing/262/95 (H1N1), and Bris07: A/Brisbane/59/2007 (H1N1). MIC: minimum inhibitory concentration. The dashed line represents the upper detection limit of MIC.

HA stem contains five hydrophobic pockets that are often targeted by the central stem antibodies (Fig. 5A)^10^. Similarly, HB31 also interacted with three of these five hydrophobic pockets (Fig. 5B) using an *IGHD3-9*-encoded LxYFxW motif in the CDR H3 (Figs. 5C, S11A), which is a recurring sequence feature of central stem antibodies^22,23^. HB31 further engaged another pocket in the upper stem (hereinafter referred to as pocket 6) (Fig. 5B). This pocket was rarely targeted by the central stem antibodies. For example, the *IGHD3-9*-encoded central stem antibodies, FI6v3 and S9-3-37, did not engage pocket 6 (Fig. S12), despite targeting the central stem epitope using the LxYFxW motif akin to HB31 (Fig. 5C). While pocket 6 is also filled by V_H_ F29 of two recently reported antibodies, 05.GC.w13.01 and 05.GC.w13.02^53^ (Fig. 5B, D), HB31 formed more substantial interaction with pocket 6. Besides filling pocket 6, V_H_ R55 of HB31 H-bonded with the backbone carbonyls of L292_HA1_ and S290_HA1_ deep inside the pocket 6 (Fig. 5D). These structural observations for HB31 also applied to HB34, which shared the same heavy chain sequence as HB31 (Fig. S2A) and displayed a binding mode almost identical to that of HB31 (Fig. 4). The inferred germline of HB31 and HB34 had a Gly at V_H_ residue 55 **(**Fig. S13A**)**. Reverting V_H_ R55 to V_H_ G55 in HB31 Fab weakened the binding affinity (K_D_) by 2-fold against A/Texas/37/2024 (H5N1) HA **(**Fig. 5E) and by more than 30% against H1 stem **(**Figs. 5E, S13B**)**. The same V_H_ R55G revertant in IgG format weakened the K_D_ against A/Texas/37/2024 (H5N1) HA and H1 stem by 5- and 4-fold, respectively (Figs. 5E, S13C). A similar observation was also made for the dissociation rate (k_off_) (Figs. 5F, S13B, C). Consistently, reverting V_H_ R55 to V_H_ G55 in HB31 reduced the neutralization potency against A/Chile/1/1983 (H1N1) and A/Beijing/262/1995 (H1N1), with the minimum inhibitory concentration (MIC) increasing from 75 μg/mL to 100 μg/mL (Fig. 5G). Furthermore, HB31 neutralized A/Michigan/45/2015 (H1N1) with an MIC of 75 μg/mL, but no neutralization activity was observed for the V_H_ R55 G revertant (Fig. 5G). These results showed that engagement of pocket 6 moderately strengthened the binding of HB31 to HA stem. Notably, pocket 6, which consists of the side chains of P293_HA1_, P306_HA1_, K307_HA1_, M59_HA2_, and T61_HA2_ (Fig. S12B), is highly conserved among H1, H2, and H5 subtypes (Fig. S12C).

## Discussion

To date, there are three major HA stem epitopes being characterized, the central stem epitope^10^, the group 1 HA-specific anchor epitope^27,54^, and the group 2 HA-specific lower stem epitope^16,17^. In this study, we showed that the HB315 epitope has a large overlap with both the central stem and the lower stem epitopes, suggesting that the three major HA stem epitopes may not be as discrete as previously thought. In addition, HB31 and HB34 target a pocket in the upper stem (pocket 6) that is rarely engaged by other known HA stem antibodies, underscoring the epitope diversity of HA stem antibodies. While peptides and small molecules targeting pockets 1-5 of the HA stem can achieve neutralization activity in the nM range^29,44^, extending those designs to target pocket 6 may further improve their potency. Together, our findings not only refine the knowledge of HA stem antigenicity but also provide structural insights into the development of next-generation therapeutics.

Although HB31, HB34, and HB315 are all encoded by *IGHV3-23*, they contain different *IGHD*-encoded motifs that are common in the CDR H3 of HA stem antibodies. HB31 and HB34 contain an *IGHD3-9*-encoded LxYFxW motif, whereas HB315 contains an *IGHD3-3*-encoded FGL motif. Consequently, the binding modes of HB31 and HB34 are different from that of HB315. This observation demonstrates that *IGHV3-23* provides a versatile framework for HA stem antibodies to adopt different *IGHD*-driven binding modes. It also suggests that at least some *IGHD* genes have a larger influence than *IGHV* genes on the binding mode of HA stem antibodies. Consistently, while *IGHV1-69* HA stem antibodies with diverse CDR H3 sequences often have similar binding modes^18^, the presence of an *IGHD3-9*-encoded LxYFxW motif changes their binding mode to become similar to that of other HA stem antibodies that possess the same motif^55^. As *IGHD*-encoded motifs have received increasing attention as recurring sequence features against different pathogens^55–58^, future studies should explore whether HA stem antibodies with *IGHD3-9*-encoded LxYFxW motif and *IGHD3-3*-encoded FG[I/V/L] motif always result in converging binding modes regardless of *IGHV* gene usage.

A notable observation in this study is that HB31 and HB34, which have minimal neutralization activity in vitro, can confer similar, if not superior, protection activity in vivo than the potently neutralizing HB315. While neutralizing antibodies are traditionally viewed as the primary mediators of antiviral protection, growing evidence indicates that antibodies with minimal to no neutralizing activity can also still confer strong in vivo protection via Fc-mediated effector functions against infections by diverse viruses, such as HIV^59,60^, SARS-CoV-2^61,62^, Zika virus^63^, Marburg virus^64^, West Nile virus^65^, and Crimean-Congo hemorrhagic fever virus^66^. For HA stem antibodies, our present study substantiates that the epitope location and approaching angle can influence Fc domain accessibility, hence the importance of Fc-mediated effector functions for protection activity in vivo. However, a recent study on HIV Env antibodies shows that their Fc-mediated effector functions are mainly determined by stoichiometry of binding and are not modulated by epitope location and approaching angle^67^. Whether the biophysical determinants for Fc-mediated effector functions are antigen-dependent warrants additional studies.

The recent shift in US funding policy against mRNA vaccine technology highlights the growing importance of broadly protective vaccine strategies for pandemic preparedness^68,69^. As exemplified during the COVID-19 pandemic, the mRNA vaccine technology has demonstrated an unprecedented ability to rapidly generate immunogens once an antigen sequence becomes available^70,71^. However, if mRNA vaccine technology becomes less accessible or deprioritized, pre-existing broad-spectrum immunity becomes more critical for pandemic preparedness. Vaccines that elicit antibodies targeting conserved epitopes, such as the influenza HA stem, offer a promising approach to mitigate the impact of unforeseeable influenza pandemic^72^. Therefore, the continued discovery and characterization of broadly protective influenza antibodies remain important, as they are key to the design of broadly protective influenza vaccines^72^.

## Methods

### Generation of phage display antibody libraries

Phage display antibody libraries were generated using a phagemid antibody library that we previously constructed from the peripheral blood mononuclear cells (PBMCs) of 245 healthy individuals with approval by the Human Research Ethics Committee at The Chinese University of Hong Kong (IRB: 2020.229)^46^. Briefly, the phagemid antibody library was electroporated into E. coli TG1 cells, expanded, and infected with M13 KO7 ΔpIII hyperphage (hyperphage) or CM13 interference-resistant helper phage (CM phage) for phage packaging. The resulting phages were precipitated, purified, and resuspended in PBS to yield the phage display antibody libraries. The phage titers were determined by trypsin treatment, bacterial infection, and colony counting.

### Panning and screening of the phage display antibody libraries

100 ng of H1 stem (in 100 µL of PBS) was added to each well of an Invitrogen Nunc MaxiSorp flat-bottom 96-well plate (Thermo Fisher Scientific), incubated overnight at 4 °C. Hyperphage and CM phage samples were normalized to give approximately 10^11^ phage per 100 µL of reaction, which was then blocked with an equal volume of 5% skim milk in PBS for 2 hours at room temperature. At the same time, wells in the Maxisorp plate were blocked with 5% skim milk (added until the brim of wells) for 2 hours at room temperature. 200 µL of blocked phage sample was added to each well and incubated for 2 hours at 37 °C. Wells were washed 5× with PBST (PBS supplemented with 0.1% Tween-20), 5× with PBS, tapped dry on a paper towel after each wash. Phage were eluted from wells with 1 µg of TPCK-treated trypsin for 30 mins at 37 °C. Eluted phage was added to 15 mL of E. coli TG1 culture outgrew to an OD_600_ of 0.45, followed by incubation at 37 °C, standing for 30 mins, and shaking for 30 mins. Transductants were concentrated by centrifuging at 4500 × *g* for 10 minutes, and the cells as well as serial dilutions were platted onto 2YT agar supplemented with 100 µg mL^−1^ of ampicillin. Plates were incubated overnight at 30 °C. Transductants were collected in 2YT medium supplemented with 30% glycerol and phage were prepared for 3 rounds of panning, as stated in the production of Fab-expressing phage. The stringency of screening was increased sequentially, by increasing the number of washes to 8× PBST + 7× PBS for the second round of panning, and 10× PBST + 10× PBS for the third round of panning.

### PacBio sequencing of the Fab libraries

The pre-selection Fab libraries and third round post-panning Fab libraries were amplified using PrimeSTAR Max DNA Polymerase (Takara Bio) per manufacturer’s instruction with the following primers (5’-GTA AAA CGA CGG CCA GTT TCA GTT TCG CTA CCG TGG CCC AAG CGG CC-3’ and 5’-CAG GAA ACA GCT ATG ACC CAC TAG TTT TTG TTC TTG GCC TGT TTG GCC ACA-3’). The PCR product was purified using a Monarch Gel Extraction Kit (New England Biolabs, cat# T1020). A second round of PCR was carried out to add the sample index sequences to the amplicons (Table S2**)**. The final PCR products were sequenced on one SMRT cell 8 M on a PacBio Revio system using the CCS sequencing mode and a 12-hour movie time.

### Analysis of PacBio sequencing data

Circular consensus sequences (CCSs) were generated from the raw subreads using SMRTLink v13.0, setting the parameters to require 99.9% accuracy and a minimum of 3 passes. CSSs in FASTQ format were parsed using the SeqIO module in BioPython and filtered based on the base calling quality score, where any read with more than five nucleotides of phred quality score <40 was removed. The adapter sequences were then identified on each read and trimmed from the Fab sequences. Reads that did not have the complete adapter sequences were also removed. The filtered reads were then aligned to the reference Fab sequences. Frequency (*F*) of Fab *i* of a given sample *s* was computed for each replicate as follows:

1 $${F}_{i,s}=\frac{{{readcount}}_{i,s}+1}{{\sum }_{s}({{readcount}}_{i,s}+1)}$$

### Cell culture

HEK293T cells (human embryonic kidney cells, female) and MDCK-SIAT1 cells (Madin-Darby canine kidney cells with stable expression of human 2,6-sialtransferase, female, Sigma-Aldrich) were cultured in Dulbecco’s modified Eagle’s medium (DMEM) with high glucose (Thermo Fisher Scientific) supplemented with 10% heat-inactivated fetal bovine serum (FBS, Thermo Fisher Scientific), 1% penicillin-streptomycin (Thermo Fisher Scientific), and 1× GlutaMax (Thermo Fisher Scientific). Cell passaging was performed every 3 to 4 days using 0.05% Trypsin-ethylenediaminetetraacetic acid (EDTA) solution (Thermo Fisher Scientific). Expi293F cells (human embryonic kidney cells, female, Thermo Fisher Scientific) were maintained in Expi293 Expression Medium (Thermo Fisher Scientific). Sf9 cells (*Spodoptera frugiperda* ovarian cells, female, ATCC) were maintained in Sf-900 II SFM medium (Thermo Fisher Scientific).

### Expression and purification of H1 stem, H3 stem, and HA

The H1 stem (#4900)^32^, H3 stem^47^, A/Solomon Island/3/2006 (H1N1) HA, A/Beijing/262/1995 (H1N1) HA, A/Michigan/45/2015 (H1N1) HA, A/Japan/305/1957 (H2N2) HA, A/Texas/37/2024 (H5N1) HA, and A/mallard/Alberta/566/1985 (H6N2) were fused with N-terminal gp67 signal peptide and a C-terminal BirA biotinylation site, thrombin cleavage site, trimerization domain, and a 6× His-tag, and then cloned into a customized baculovirus transfer vector^16^. Subsequently, recombinant bacmid DNA was generated using the Bac-to-Bac system (Thermo Fisher Scientific) according to the manufacturer’s instructions. Baculovirus was generated by transfecting the purified bacmid DNA into adherent Sf9 cells using Cellfectin reagent (Thermo Fisher Scientific, cat# 10362100) according to the manufacturer’s instructions. The baculovirus was further amplified by passaging in adherent Sf9 cells at a multiplicity of infection (MOI) of 1. Recombinant proteins were expressed by infecting 1 L of suspension Sf9 cells at an MOI of 1. On day 3 post-infection, Sf9 cells were pelleted by centrifugation at 4000 ×*g* for 25 min, and soluble recombinant proteins were purified from the supernatant by affinity chromatography using Ni Sepharose Excel resin (Cytiva) and then size exclusion chromatography using a HiLoad 16/100 Superdex 200 prep grade column (Cytiva) in 20 mM Tris-HCl, pH 8.0, 100 mM NaCl. The purified proteins were concentrated by an Amicon spin filter (Millipore Sigma) and filtered by 0.22 µm centrifuge tube filters (Costar). The concentration of each protein sample was determined by nanodrop (Fisher Scientific). Proteins were subsequently aliquoted, flash frozen by dry-ice ethanol mixture, and stored at −80 °C until used.

The following proteins were obtained from BEI Resources: A/California/07/2009 (H1N1) HA (NR-42635), A/bald eagle/Florida/W22–134-OP/2022 (H5N1) HA (NR-59476), A/northern pintail/WA/40964/2014 (H5N8) HA (NR-50174), A/chicken/Netherlands/14015531/2014 (H5N8) HA (NR-50133), and A/Hong Kong/33982/2009 (H9N2) HA (NR-41792). The following proteins were obtained from Sino Biological: A/USSR/90/1977 (H1N1) HA, A/Taiwan/01/1986 (H1N1), and A/Brisbane/02/2018 (H1N1) HA.

### Expression and purification of IgG and Fab

The heavy and light chain genes of each antibody were synthesized as eBlocks (Integrated DNA Technologies), and then cloned into human IgG1 or murine IgG2c and human or murine light chain (kappa or lambda) expression vectors using Gibson assembly according to a previously described method^73^. The plasmids of heavy chain and light chain were transiently co-transfected into Expi293F cells at a mass ratio of 2:1 (HC:LC) using ExpiFectamine 293 Reagent (Thermo Fisher Scientific, cat# A14525). After transfection, the cell culture supernatant was collected at 6 days post-transfection. The IgG and Fab were then purified using a CaptureSelect CH1-XL pre-packed column (Thermo Fisher Scientific).

### Enzyme-linked immunosorbent assay (ELISA)

Nunc MaxiSorp ELISA plates (Thermo Fisher Scientific) were utilized and coated with 100 μL of recombinant proteins at a concentration of 1 μg ml^−1^ in a 1× PBS solution. The coating process was performed overnight at 4 °C. On the following day, the ELISA plates were washed three times with 1× PBS supplemented with 0.05% Tween 20, and then blocked using 200 μL of 1× PBS with 5% non-fat milk powder for 2 hours at room temperature. After the blocking step, 100 μL of IgGs from the supernatant were added to each well and incubated for 2 hours at 37 °C. The ELISA plates were washed three times to remove any unbound IgGs. Next, the ELISA plates were incubated with horseradish peroxidase (HRP)-conjugated goat anti-human IgG antibody (1:5000, Invitrogen) for 1 hour at 37 °C. Subsequently, the ELISA plates were washed five times using PBS containing 0.05% Tween 20. Then, 100 μL of 1-Step Ultra TMB-ELISA Substrate Solution (Thermo Fisher Scientific) was added to each well. After 15 min incubation, 50 μL of 2 M H_2_SO_4_ solution was added to each well. The absorbance of each well was measured at a wavelength of 450 nm using a BioTek Synergy HTX Multimode Reader.

### Binding competition assay

Binding competition assays were performed by biolayer interferometry (BLI) using an Octet Red96e instrument (Sartorius) at room temperature, with modifications to a previously described method^74^. Briefly, His-tagged HA protein at 0.5 μM in 1× kinetics buffer (1× PBS, pH 7.4, 0.01% w/v BSA and 0.002% v/v Tween 20) was loaded onto anti-Penta-HIS (HIS1K) biosensors. The assay consisted of five steps: (1) baseline: 60 s with 1× kinetics buffer; (2) loading: 480 s with His-tagged HA protein; (3) baseline: 60 s with 1× kinetics buffer; (4) association: 120 s with 200 nM antibody A (first antibody) in Fab format or with buffer; and (5) dissociation: 120 s with a mix of 200 nM antibody A (first antibody) and 200 nM antibody B (second antibody) in Fab format. For a given antibody B, its percentage competition with antibody A was computed as follows:

2 $$\%\,{competition}=\left(1-\frac{{response}\,{of}\,{antibody}\,B\,{with}\,{Fab}\,A\,{prebound}}{{response}\,{of}\,{antibody}\,B\,{without}\,{Fab}\,A\,{prebound}}\right)\times 100$$

### Recombinant virus construction and purification

Recombinant A/Puerto Rico/8/1934 (H1N1) (PR8) virus was rescued using the eight-plasmid influenza virus reverse genetics system^75^. Additionally, we rescued 7:1 reassortant viruses by using the HA segments from A/Chile/1/1983 (H1N1), A/Beijing/262/1995 (H1N1), A/Brisbane/59/2007 (H1N1), A/California/07/09 (H1N1), A/Michigan/45/2015 (H1N1), A/England/234600203/2023 (H1N2) and A/Vietnam/1203/2004 (H5N1) with the other seven segments from PR8 as previously described^76^. Briefly, DNA plasmids were transfected into a co-culture of HEK293T cells and MDCK-SIAT1 cells (ratio of 6:1) using Lipofectamine 2000 (Thermo Fisher Scientific, cat# 11668500) and incubated at 37 °C for 48 hours. Recombinant viruses in the supernatant were plaque-purified on MDCK-SIAT1 cells. Individual plaques were picked and incubated with fresh MDCK-SIAT1 cells, and viral RNAs were extracted from supernatant at 72 hours post-infection. The sequences of HA segments were confirmed by Sanger sequencing. All experiments involving H5 viruses were performed in the Biosafety Level 3 (BSL3) facility at The Chinese University of Hong Kong.

### Virus neutralization assay

MDCK-SIAT1 cells were seeded in a 96-well, flat-bottom cell culture plate (Thermo Fisher Scientific). The next day, serially diluted monoclonal antibodies were mixed with an equal volume of virus and incubated at 37 °C for 1 hour. The antibody-virus mixture was then incubated with MDCK-SIAT1 cells at 37 °C for 1 hour after the cells were washed twice with PBS. Subsequently, the antibody-virus mixture was replaced with Minimum Essential Medium (MEM) supplemented with 25 mM of 4-(2-hydroxyethyl)−1-piperazineethanesulfonic acid (HEPES) and 1 μg mL^−1^ of Tosyl phenylalanyl chloromethyl ketone (TPCK)-trypsin. The plate was incubated at 37 °C for 72 hours and the presence of virus was detected by hemagglutination assay. The results were analyzed using Prism software (GraphPad).

### ADCC reporter bioassay

1.5 × 10^4^ MDCK cells were seeded in white, flat-bottom 96-well cell culture plates (Thermo Fisher) and incubated at 37 °C overnight. The next day, cells were washed three times with PBS, and 100 μL of PR8 virus at 1.5 × 10^6^ plaque forming units (PFU) mL^−1^ in MEM was added to each well. After incubating the plate for 24 hours at 37 °C, the media was removed, and 25 μL of serially diluted antibodies in 1:10 in Roswell Park Memorial Institute (RPMI) 1640 media (Thermo Fisher), 25 μL of effector cells (engineered Jurkat cells stably expressing human FcγRIIIa V158 and NFAT-induced luciferase) and 25 μL of RPMI 1640 media were added. After 6 hours of incubation at 37 °C, 75 μL of Bio-Glo luciferase (Promega, cat# G7940) was added to each well. The plate was incubated for 10 min in the dark and the luciferase-induced luminescence was measured with a BioTek Synergy HTX Multimode Reader (Agilent). Fold titer increase of antibody *i* at concentration *c* was calculated as follow:

3 $${{Fold}\,{titer}\,{increase}}_{i,c}=\frac{{{Luciferase}\,{signal}}_{i,c}}{{mean}\left({{Luciferase}\,{signal}}_{{IgG}\,{control}}\right)}$$

Data were analyzed using Prism software, and the area under the curve (AUC) values were determined for log(fold titer increase) versus log(antibody concentration).

### Prophylactic and therapeutic protection experiments

Per experimental group, a total of 8 female BALB/c mice at 6 weeks old were anesthetized with isoflurane and intranasally infected with 5× median lethal dose (LD_50_) of PR8 H1N1 virus or PR8-H5N1 (7:1 on backbone from A/Puerto Rico/8/1934). Mice were given the indicated antibody at a dose of 5 mg kg^−1^ intraperitoneally at 4 hours before infection (prophylaxis) or 24 hours after infection (therapeutics). Weight loss was monitored daily for 14 days in 5 out of 8 mice per experimental group. The humane endpoint was defined as a weight loss of 25% from initial weight on day 0. To determine the lung viral titers on day 3 post-infection, lungs were harvested from the remaining three mice in each experimental group and homogenized in 1 mL of MEM with 1 μg mL^−1^ of TPCK-trypsin with gentleMACS Dissociator (Miltenyi Biotec). Subsequently, viral titers were measured by TCID_50_ (median tissue culture infectious dose) assay. The results were analyzed using Prism software. The animal experiments were performed in accordance with protocols approved by UIUC Institutional Animal Care and Use Committee (IACUC). All mouse experiments involving H5 viruses were conducted in the Biosafety Level 3 (BSL3) facility at The Chinese University of Hong Kong. Of note, mice were humanely euthanized by carbon dioxide (CO₂) inhalation using a gradual-fill method in accordance with institutional animal care and use guidelines. Death was confirmed by a secondary physical method (cervical dislocation) before tissue collection.

### Cryo-EM sample preparation and data collection

The purified A/Solomon Islands/3/2006 (H1N1) HA protein was mixed with the Fab at a 1:4 molar ratio (one HA trimer per four Fabs) and incubated overnight at 4 °C before purifying by size exclusion chromatography on the Superose 6 Increased 10/300 column (Cytiva) in 20 mM Tris-HCl, pH 8.0, and 100 mM NaCl. For the complexes with HB31 and HB34, anchor Fab FISW84^54^ was also added to reduce the preferred orientation. The complex peak was concentrated to ~3 mg mL^−1^ and mixed with 0.5% w/v n-octyl-ß-D-glucoside (Anagrade) to a final concentration of 0.1% w/v just before loading on the grid. Cryo-EM grids were prepared using a Vitrobot Mark IV machine. An aliquot of 3 µL sample was applied to a 300-mesh Quantifoil R1.2/1.3 Cu grid pretreated with glow-discharge. High-resolution cryo-EM movies were collected on an FEI Titan Krios at 300 kV with a Gatan K3 detector.

### Cryo-EM image processing and model building

Data processing was performed with CryoSPARC (version 4.5)^77^. Movies were subjected to motion correction and CTF estimation, and particles were picked with the CryoSPARC blob picker followed by 2D classification. Best classes from blob picker were used as templates for CryoSPARC template pickers, and the resulting particles were cleaned up by multiple rounds of 2D classification before ab initio reconstruction. The best class from ab initio reconstruction was subjected to homogenous refinement, reference-based motion correction, another round of homogenous refinement, local and global CTF estimation, and non-uniform refinement. All datasets except for HB31 were processed with C3 symmetry. Due to substoichiometric quantities of bound FISW84 Fab in the HB31 complex, which was likely attributed to the weak binding affinity of FISW84 Fab, the data were processed in C1 symmetry, and FISW84 Fab was not built into the map. FISW84 Fab was utilized exclusively as a tool to address the preferred orientation issues encountered during cryo-EM grid preparation of the HA-HB31 complex. Its addition to the complex potentially changed the physical and electrostatic properties of the complex, thereby facilitating more diverse particle orientations in the ice. Because FISW84 is a well-characterized antibody with a previously published structure (PDB ID: 6HJP)^54^, and since it binds to a distinct anchor epitope that does not overlap with the HB31 stem epitope, its partial occupancy did not interfere with the high-resolution modeling of the HA-HB31 interface. The density for the HA trimer and the HB31 Fabs of interest remained clear and robust. As FISW84 was not the subject of our investigation, further optimization of its binding kinetics was deemed outside the scope of this structural study. All maps were sharpened with DeepEMhancer^78^, and all initial atomic models were built using ModelAngelo^79^. The models were subjected to multiple rounds of manual refinement in Coot (version 0.9.8)^80^ and real-space refinement in Phenix^81^. This process was iterated for several cycles until no significant improvement of the model was observed. The buried surface area (BSA) of the epitope and paratope was computed using PDBePISA^82^.

### Antibody binding angle and height measurement

To measure antibody binding angle and height within each HA-antibody complex, we represented the HA stem as a vector with the lower point of the vector set at the origin. The variable domain of each Fab was aligned to CR8020, which served as a reference Fab, to normalize the position of the constant domain. We then defined a Fab vector between the center of mass of the complex’s variable domain (COM_var_) and the center of mass of the reference constant domain. The binding angle (α) was calculated between this vector and the HA stem, while the z-height (z) was defined as the z-coordinate of COM_var_.

### Biolayer interferometry binding assay

Binding assays were performed by biolayer interferometry (BLI) using an Octet Red96e instrument (Sartorius) at room temperature. Briefly, the indicated Fabs at 300 nM, 100 nM, and 33 nM in 1× kinetics buffer (PBS at pH 7.4, 0.01% w/v BSA, and 0.002% v/v Tween 20) were loaded onto Anti-Human Fab-CH1 2nd Generation (FAB2G) biosensors and incubated with A/Texas/37/2024 (H5N1) HA and H1 stem at 20 µg mL^−1^. The assay consisted of five steps, each run for at least 60 seconds: (1) baseline: 1× kinetics buffer; (2) loading: Fab proteins; (3) baseline: 1× kinetics buffer; (4) association: HA proteins; and (5) dissociation: 1× kinetics buffer. For estimating the K_D_, a 1:1 binding model was used.

### Thermal shift assay

Thermal shift assay was performed using differential scanning fluorimetry (DSF) on the CFX Opus 96 Real-Time PCR System (Bio-Rad, cat# 12011319). SYPRO Orange Protein Gel Stain (5,000X Concentrate in DMSO) (Thermo Fisher Scientific, cat# S6650). For each protein sample, 5 μg was used per assay in a total volume of 25 µL with SYPRO Orange Protein Gel Stain at 1X in PBS in a Hard-Shell 96-Well PCR Plate (Bio-Rad, cat# HSP9601). The assay was performed from 10 °C to 95 °C in increments of 0.5 °C for 10 s plus plate reading using the FRET channel of the instrument.

### Sequence analysis

A total of 6948 HA sequences from different influenza A subtypes were downloaded from GISAID^83^. Sequence alignment was performed using MAFFT (v7.526)^84^. Sequence logos were created using Logomaker^85^.

### Reporting summary

Further information on research design is available in the Nature Portfolio Reporting Summary linked to this article.

## Supplementary information

Supplementary Information

Reporting Summary

Transparent Peer Review file

## Source data

Source Data

## Data Availability

PacBio sequencing data generated in this study have been submitted to the NIH Short Read Archive under accession number: BioProject PRJNA1104662. Cryo-EM maps have been deposited to the Electron Microscopy Data Bank under accession codes: EMD-70809, EMD-70810, and EMD-70811. The refined models have been deposited to the RCSB Protein Data Bank under accession codes 9OSS, 9OST, and 9OSU. All other data are available in the article and its Supplementary files or from the corresponding author upon request. Source data are provided with this paper.

## Code availability

Custom scripts for analyzing the PacBio sequencing data have been deposited to https://zenodo.org/records/20435345^86^.
