# Supplementary material for: Structural basis for distinct protective mechanisms of IGHV3-23 antibodies targeting influenza hemagglutinin stem
Source: Nat Commun. 2026 Jun 22;17:7822. doi: 10.1038/s41467-026-74658-w (PMC13438699; doi:10.1038/s41467-026-74658-w)
Supplement: Supplementary file 1 [file 41467_2026_74658_MOESM1_ESM.pdf]

Table S1. Cryo-EM data collection, refinement and validation statistics.

	HB31 Fab + SI06 HA + FISW84 Fab	HB34 Fab + SI06 HA + FISW84 Fab	HB315 Fab + SI06 HA
**Data collection and processing**			
Magnification	81,000	81,000	81,000
Voltage (kV)	300	300	300
Electron exposure (e^-/Å^2)	57.35	57.35	57.35
Defocus range (μm)	-0.5 – 3.0	-0.5 – 3.0	-0.5 – 3.0
Pixel size (Å)	0.529	0.529	0.529
Symmetry imposed	C1	C3	C3
Initial particle images (no.)	599,816	714,635	647,100
Final particle images (no.)	406,141	304,985	300,983
Map resolution (Å)	2.76	2.64	2.67
FSC threshold 0.143			
Map postprocessing	DeepEMhancer	DeepEMhancer	DeepEMhancer
**Refinement**			
Initial model used (PDB code)	N/A	N/A	N/A
Model composition			
Non-hydrogen atoms	16,352	20,816	16,181
Protein residues	2,161	2,753	2,154
Root-mean-square deviations			
Bond lengths (Å)	0.003	0.003	0.004
Bond angles (°)	0.526	0.547	0.581
Validation			
MolProbity score	1.95	2.05	2.15
Clashscore	7.19	6.34	5.89
Poor rotamers (%)	2.04	3.00	4.45
Ramachadran plot			
Favored (%)	95.35	95.17	95.12
Allowed (%)	4.51	4.83	4.84
Disallowed (%)	0.14	0.00	0.05
**PDB code**	9OSS	9OST	9OSU
**EMDB code**	EMD-70809	EMD-70810	EMD-70811

Table S2. Sequences of sample indexing primers.

Primer name	Sequence (5' to 3')
PacBio-F-1	TGAACCTTGTAAAACGACGGCCAGTTTCAG
PacBio-F-2	TGCTAAGTGTAAAACGACGGCCAGTTTCAG
PacBio-F-3	TGTTCTCTGTAAAACGACGGCCAGTTTCAG
PacBio-R-1	GTCGTGATCAGGAAACAGCTATGACCCACT
PacBio-R-2	ACCACTGTCAGGAAACAGCTATGACCCACT
PacBio-R-3	TGGATCTGCAGGAAACAGCTATGACCCACT

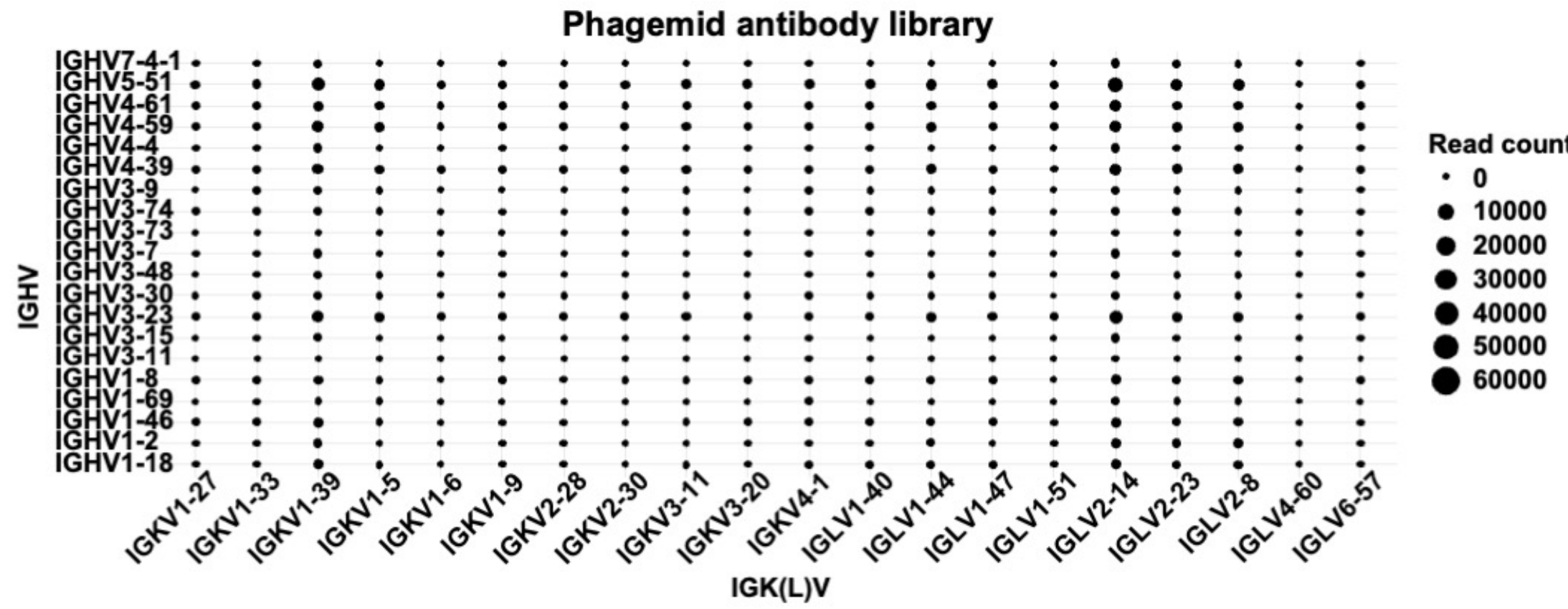

Figure S1. Distribution of IGHV/IGK(L)V pairings in the phagemid antibody library. Point size reflects the sequencing read count for each IGHV/IGK(L)V combination.

A

Heavy chain variable domain

```
                                           CDR H1                      CDR H2
HB31    EVQLLESGGGLVQPGGSLRLSCTVSGFTSSNYDMSWVRQAPGKGLEWVSGI-SGTGRDTYYADSVKGR
HB34    EVQLLESGGGLVQPGGSLRLSCTVSGFTSSNYDMSWVRQAPGKGLEWVSGI-SGTGRDTYYADSVKGR
HB315   EVQLLESGGGLAQPGGSLRLSCTASGFTFSNFAMNWVRQAPGKGLEWVSFINSGSG-STYYADSVKGR

                                                 CDR H3
HB31    FTISRDNSKNTLSLQMNSLRADDTAVYFCARD--RPVLRYFDWQPYGL-------DVWGKGTTVTVSS
HB34    FTISRDNSKNTLSLQMNSLRADDTAVYFCARD--RPVLRYFDWQPYGL-------DVWGKGTTVTVSS
HB315   FTISRDNSENTLFLQMNSLRAEDTAVYYCAKDLGRAI--------FGLVIPEGAFDIWGQGTMVTVSS
```

B

Light chain variable domain

```
                                     CDR L1                   CDR L2
HB31    QSALTQ-PPSASGSPGQSVTISCTGTSSDVGGYNYVSWYQQYPSKAPKLMIYEVSKRP
HB34    QSALTQ-PASVSGSPGQSITISCTGTSSDVGSYNLVSWYQQHPGKAPKLMIYEGSQRP
HB315   EIVMTQSPSSLSASVGDRVTITCRASQS---INNHLNWYQQKPGKAPKLLIYAASSLQ

                                             CDR L3
HB31    SGVPDRFSGSKSGNTASLTVSGLQAEDEADYYCNSYAGSNLYVFGTGTKVTVL
HB34    SGVSNRFSGSKSGNTASLTISGLQAEDEADYYCCSYASSTSYVFGTGTKVTVL
HB315   SGVPSRFSGSGSGTDFTLTISSLQPADFATYHCQQSYGVPL-TFGQGTKVEIK
```

Figure S2. Sequence alignment of HB31, HB34, and HB315. Sequence alignment was performed using MAFFT (v7.526)[1]. Residues that differ from HB31 are colored in red. **(A)** Heavy chain variable domain. **(B)** Light chain variable domain.

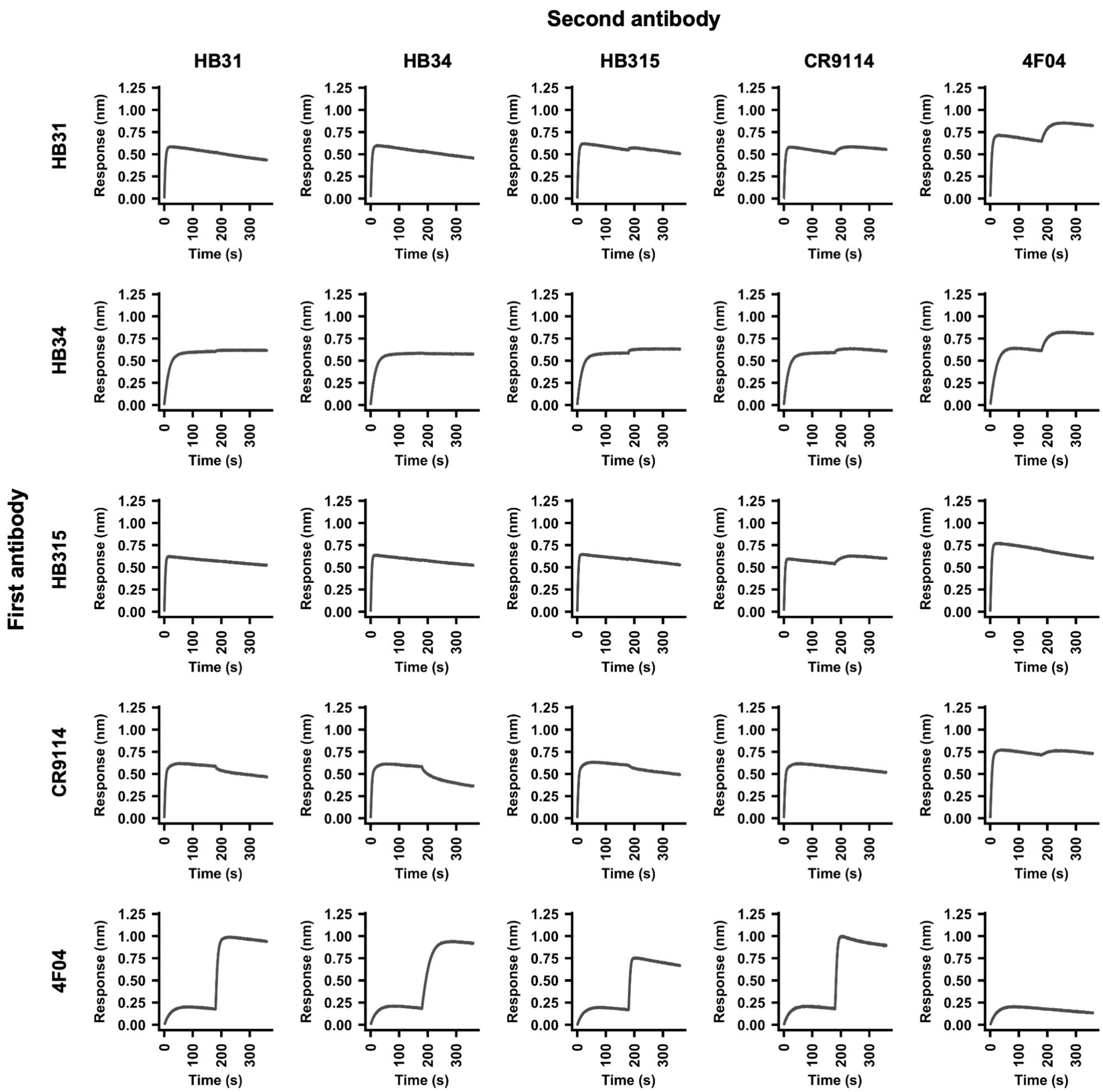

Figure S3. Sensorgrams of the antibody competition assay. Competition between antibodies for binding to A/Michigan/45/2015 (H1N1) HA was determined by biolayer interferometry. Sensorgrams represent the association step of the first antibody (200 nM, 180 s), followed by incubation with a mixture of the first and second antibodies (both at 200 nM) for an additional 180 s. All antibodies were in Fab format. Of note, CR9114 and 4F04 bind to the central stem epitope[2] and anchor epitope[3,4], respectively.

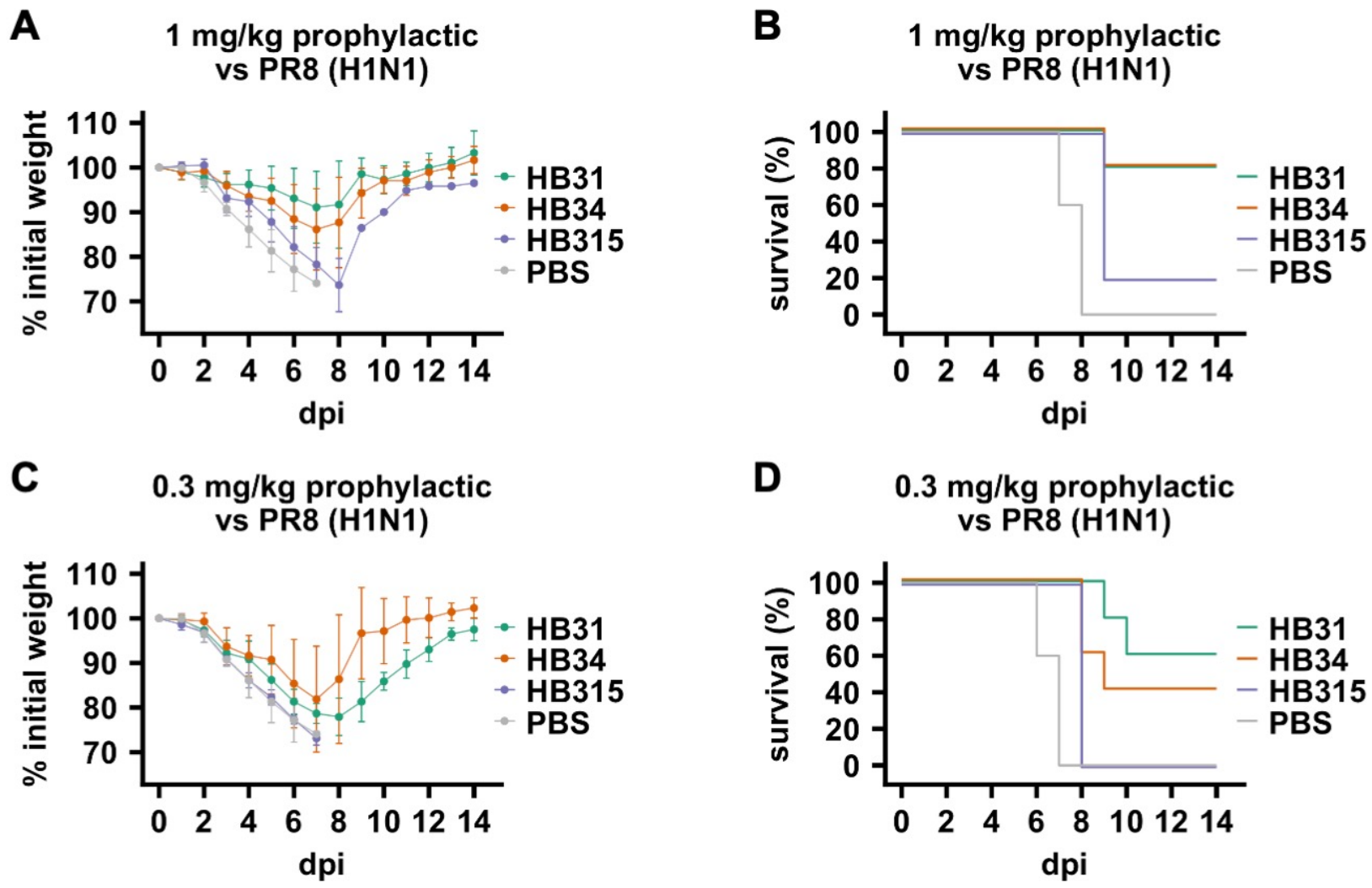

Figure S4. *In vivo* protection activity of HB31, HB34, and HB315 at 1 mg/kg and 0.3 mg/kg. Female BALB/c mice at 6 weeks old were injected intraperitoneally with the indicated antibodies at the indicated dose 4 hours prior to challenge with 5× LD_{50} of A/Puerto Rico/8/1934 (H1N1). **(A and C)** The mean percentage of body weight change post-infection is shown (n = 5 per group). The humane endpoint was defined as a weight loss of 25% from initial weight on day 0. **(B and D)** Kaplan-Meier survival curves are shown (n = 5 per group).

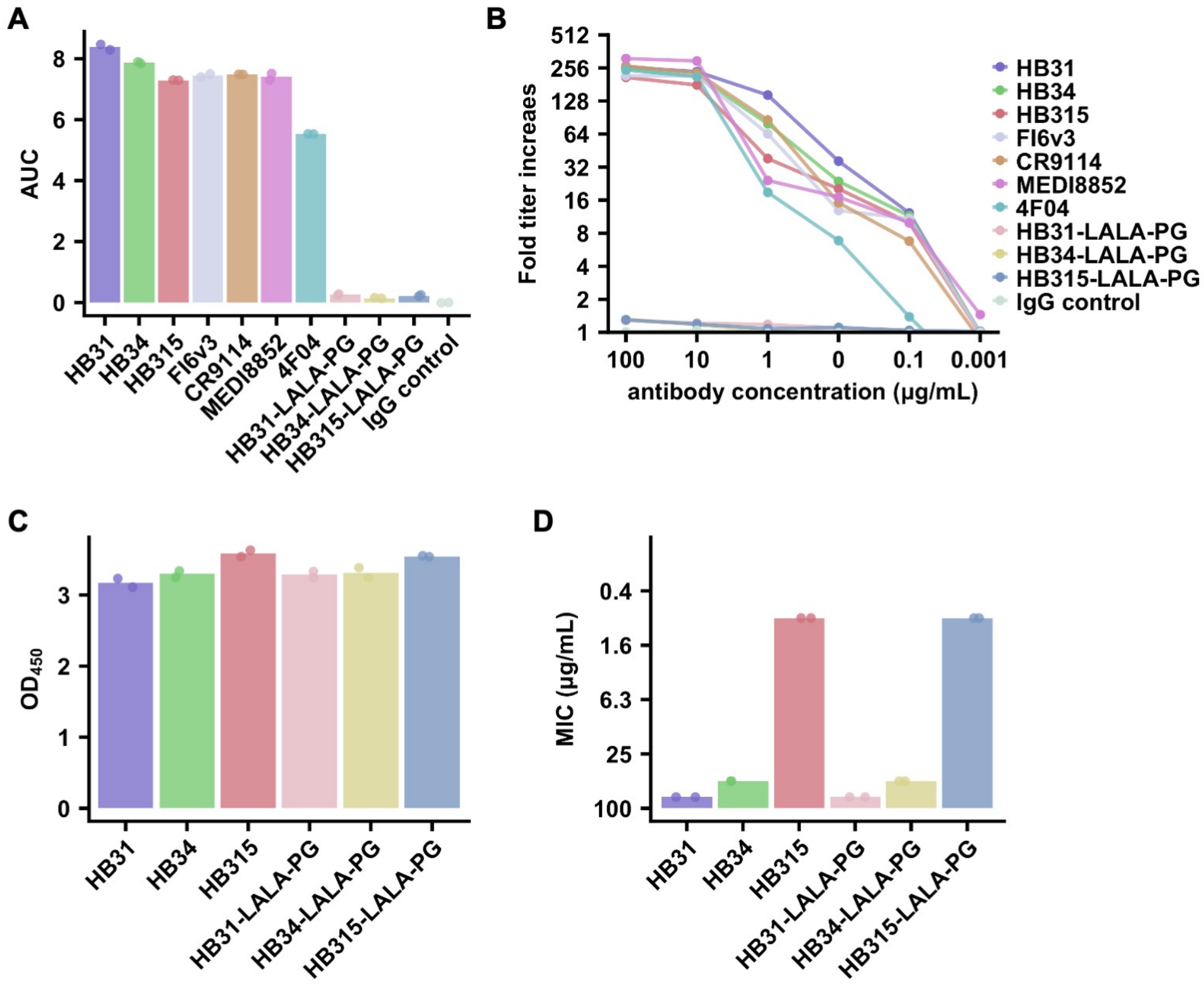

Figure S5. Antibody-dependent cellular cytotoxicity (ADCC) report assay and characterization of LALA-PG variants. (A-B) ADCC activity of the indicated antibodies was measured. FI6v3[5], CR9114[2], MEDI8852[6] are central stem antibodies, whereas 4F04[3,4] is an anchor antibody. FI6v3, CR9114 and MEDI8852 have previously been shown to exhibit ADCC activity[7,8], and therefore served as positive controls. IgG from human serum served as a negative control (IgG control). **(A)** Area under the curve (AUC) and **(B)** titration curves are shown.

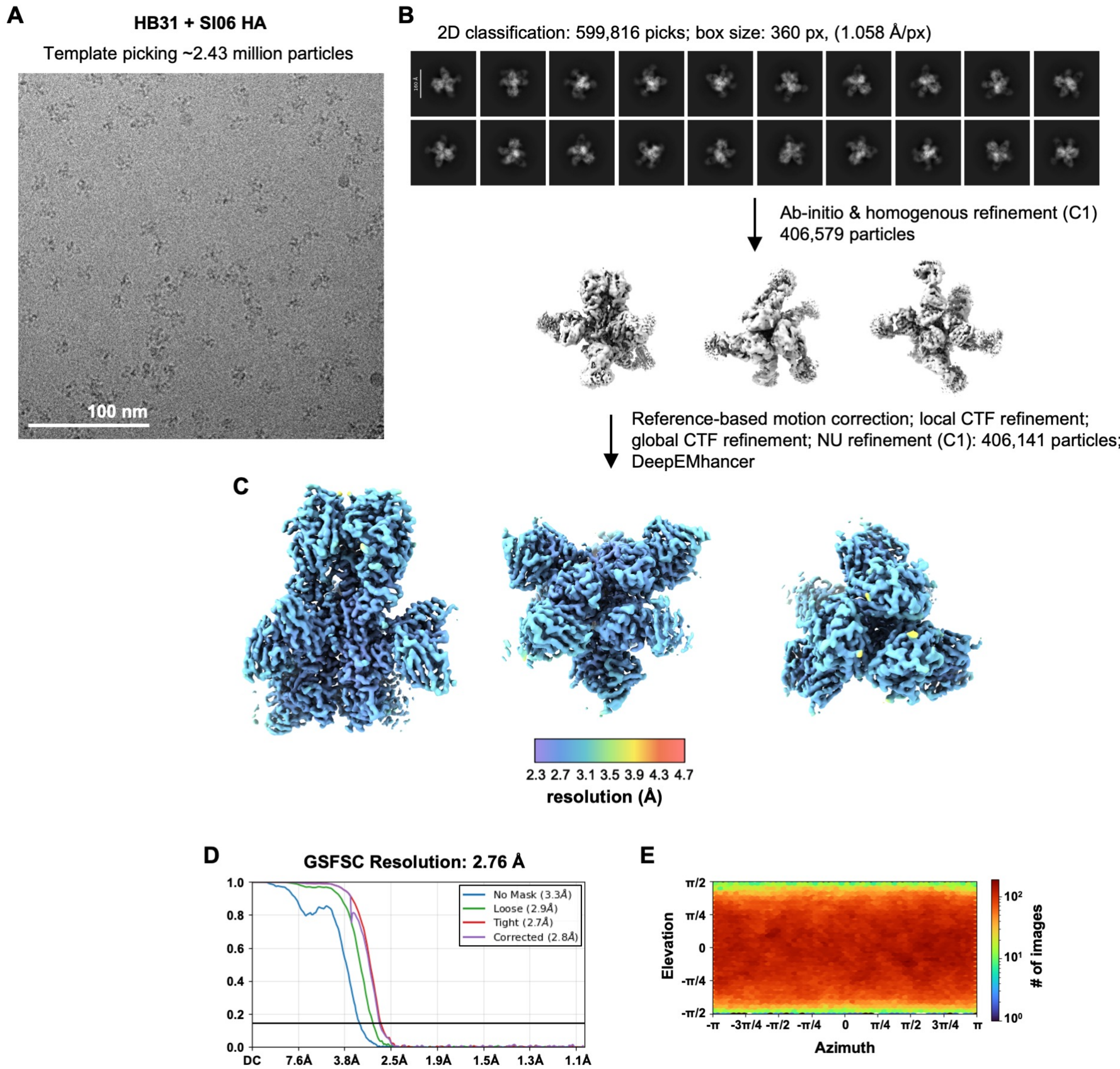

Figure S6. Cryo-EM data processing for the structure of HB31 in complex HA. (A) A representative micrograph, **(B)** representative 2D class averages and representative views of the 3D volumes from homogenous refinement, **(C)** multiple views of sharpened 3D volume from non-uniform refinement with local resolutions, **(D)** Fourier shell correlation (FSC) curves are shown, and **(E)** Azimuth plot are shown.

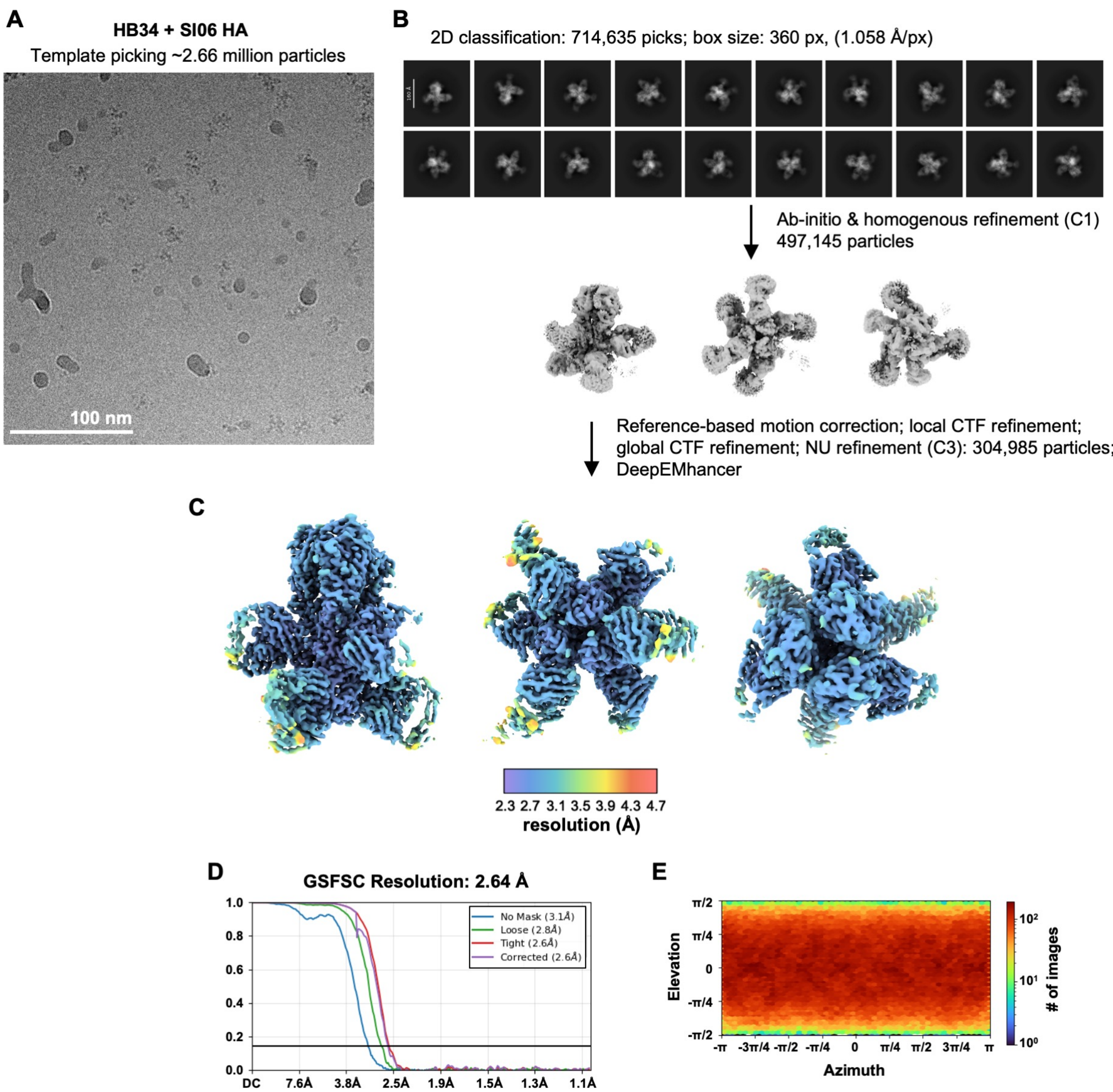

Figure S7. Cryo-EM data processing for the structure of HB34 in complex HA. (A) A representative micrograph, **(B)** representative 2D class averages and representative views of the 3D volumes from homogenous refinement, **(C)** multiple views of sharpened 3D volume from non-uniform refinement with local resolutions, **(D)** Fourier shell correlation (FSC) curves are shown, and **(E)** Azimuth plot are shown.

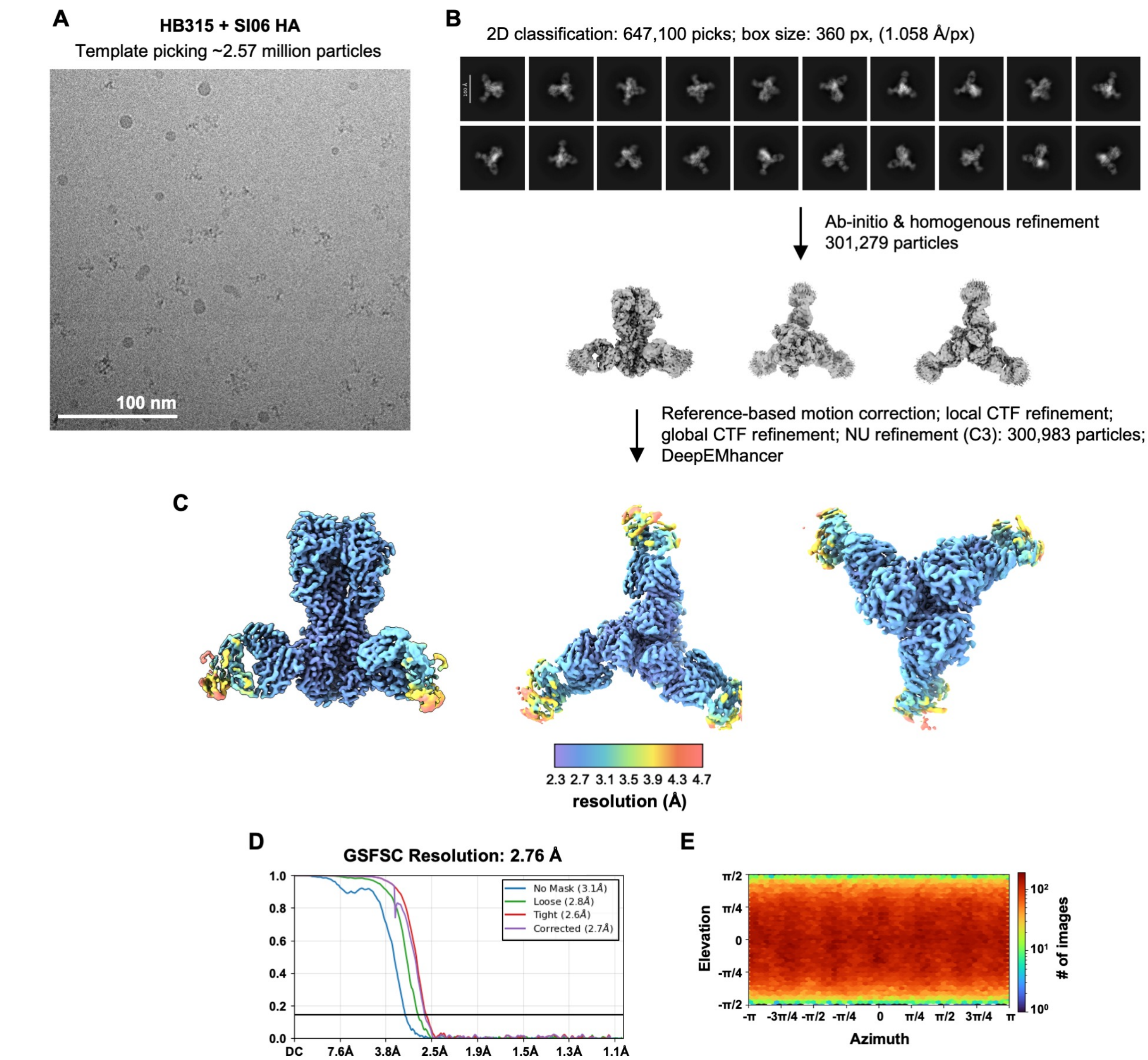

Figure S8. Cryo-EM data processing for the structure of HB315 in complex HA. (A) A representative micrograph, **(B)** representative 2D class averages and representative views of the 3D volumes from homogenous refinement, **(C)** multiple views of sharpened 3D volume from non-uniform refinement with local resolutions, **(D)** Fourier shell correlation (FSC) curves are shown, and **(E)** Azimuth plot are shown.

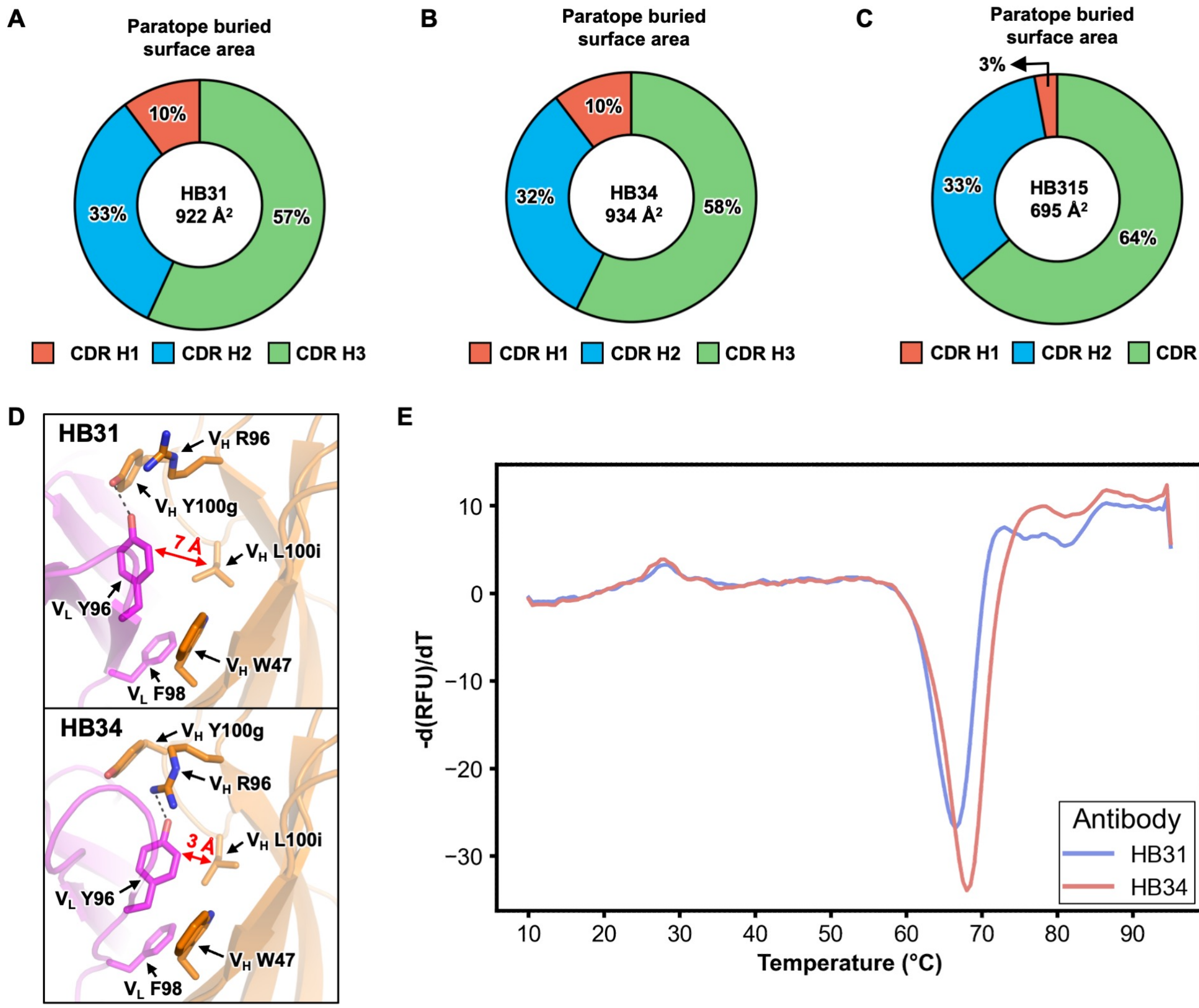

Figure S9. Structural analysis of HB31, HB34, and HB315 and thermostability of HB31 and HB34. Contributions of different CDRs of **(A)** HB31, **(B)** HB34, and **(C)** HB315 to the paratope buried surface area are shown. **(D)** The V_H-V_L interfaces of HB31 (upper panel) and HB34 (lower panel) are shown. H-bonds are shown black dashed lines. Heavy chain variable domain (V_H) and light chain variable domain (V_L) are in orange and magenta, respectively. Distance of the nearest atoms between V_H L100i and V_L Y96 is labeled in red. **(E)** Thermostability of HB31 and HB34 was measured by thermal shift assay. The first differential curves for the relative fluorescence unit (RFU) with respect to temperature are shown for HB31 (blue) and HB34 (red).

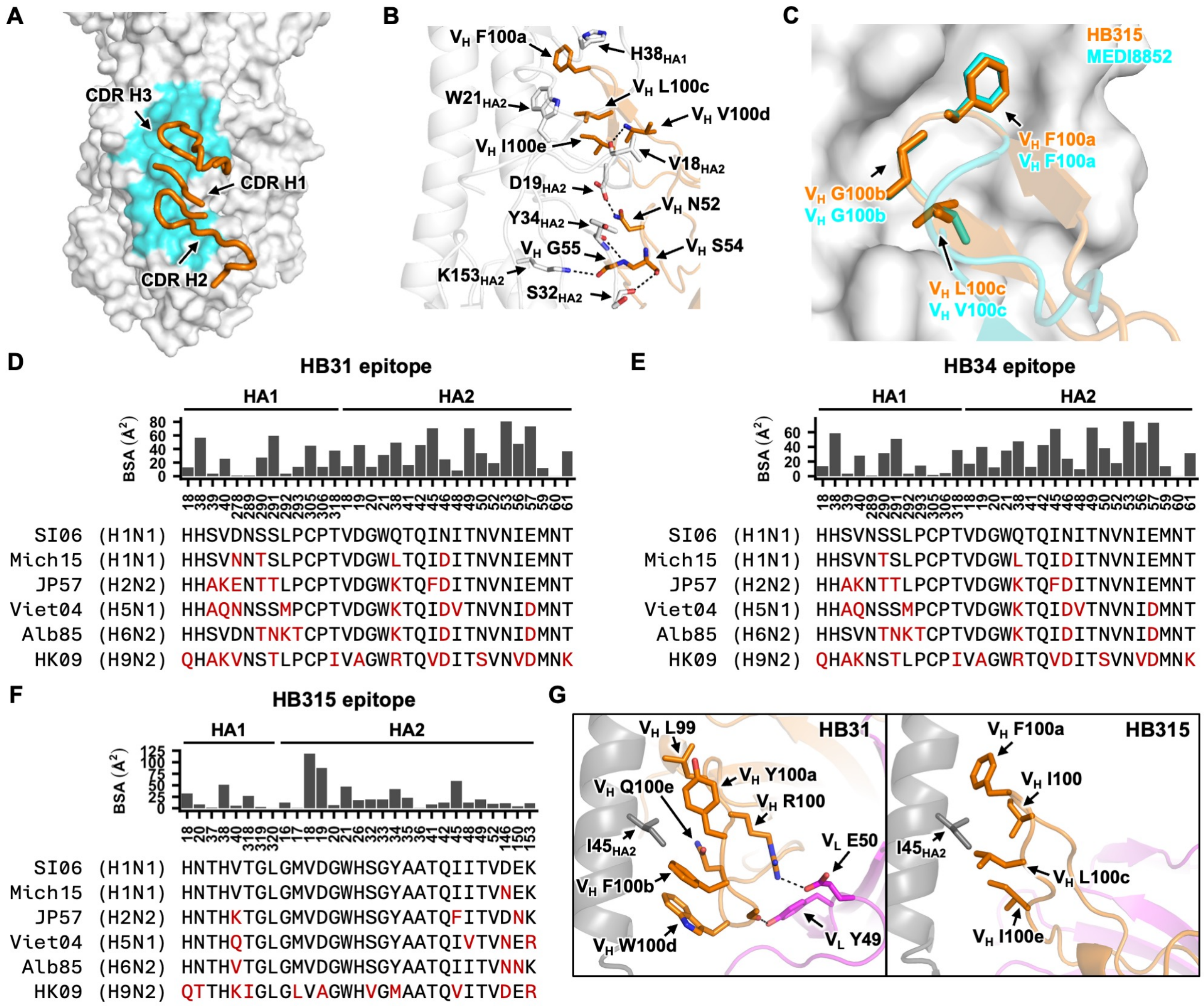

Figure S10. Epitope analysis. (A) HA is shown as surface representation with the HB315 epitope colored in cyan. The heavy chain CDRs of HB315 are shown as orange cartoon. **(B)** Key residues at the interface between HA and HB315 are shown. H-bonds are shown black dashed lines. **(C)** Interactions between HA (white surface) and the FG[L/V] motifs in the CDR H3s of MEDI8852 (PDB 5JW4, cyan)[6] and HB315 (orange) are shown. **(D-F)** The buried surface area (BSA) of each epitope residue upon binding to **(D)** HB31, **(E)** HB34, and **(F)** HB315 is shown. The amino acid sequence of the indicated strains for each epitope residue is shown at the bottom. Red indicates sequence differences from A/Solomon Island/3/2006 (H1N1) HA. SI06: A/Solomon Island/3/2006 (H1N1), Mich15: A/Michigan/45/2015 (H1N1), JP57: A/Japan/305/1957 (H2N2), Viet04: A/Vietnam/1203/2004 (H5N1), Alb85: A/mallard/Alberta/566/1985 (H6N2), and HK09: A/Hong Kong/33982/2009 (H9N2). **(G)** Interaction between $I45_{HA2}$ and HB31 or HB315 are shown. H-bonds and electrostatic interactions are shown black dashed lines. Heavy chain and light chain are in orange and magenta, respectively.

A

HB31/HB34

```
                     C  A  R  D  R  P  V  L  R  Y  F  D  W  Q  P  Y  G  L  D  V  W
               HB31:TGTGCGAGAGATCGCCCCGTGTTACGATATTTTGACTGGCAACCATACGGTTTGGACGTCTGG
  Germline sequence:TGTGCGAAA         GTGTTACGATATTTTGACTGG    TACGGTTTGGACGTCTGG
                    IGHV3                 IGHD3-9                   IGHJ6
```

B

HB315

```
                     C  A  K  D  L  G  R  A  I  F  G  L  V  I  P  E  G  A  F  D  I  W
               HB31:TGTGCGAAAGACTTAGGGCGGGCGATTTTTGGACTGGTTATCCCAGAGGGTGCTTTTGATATCTGG
  Germline sequence:TGTGCGAAAGA         CGATTTTTGGACTGGTTAT          TGCTTTTGATATCTGG
                    IGHV3                  IGHD3-3                      IGHJ3
```

Figure S11. Putative germline sequences for the CDR H3s of HB31, HB34, and HB315**.** Amino acid and nucleotide sequences of the heavy chain V-D-J junction are shown for **(A)** HB31, HB34, and **(C)** HB315. Putative germline sequences and segments for IGHV, IGHD, and IGHJ, are indicated and colored in blue, purple, and red, respectively. The grey shading indicates a somatically mutated nucleotide. Intervening spaces at the V-D and D-J junctions are N-nucleotide additions.

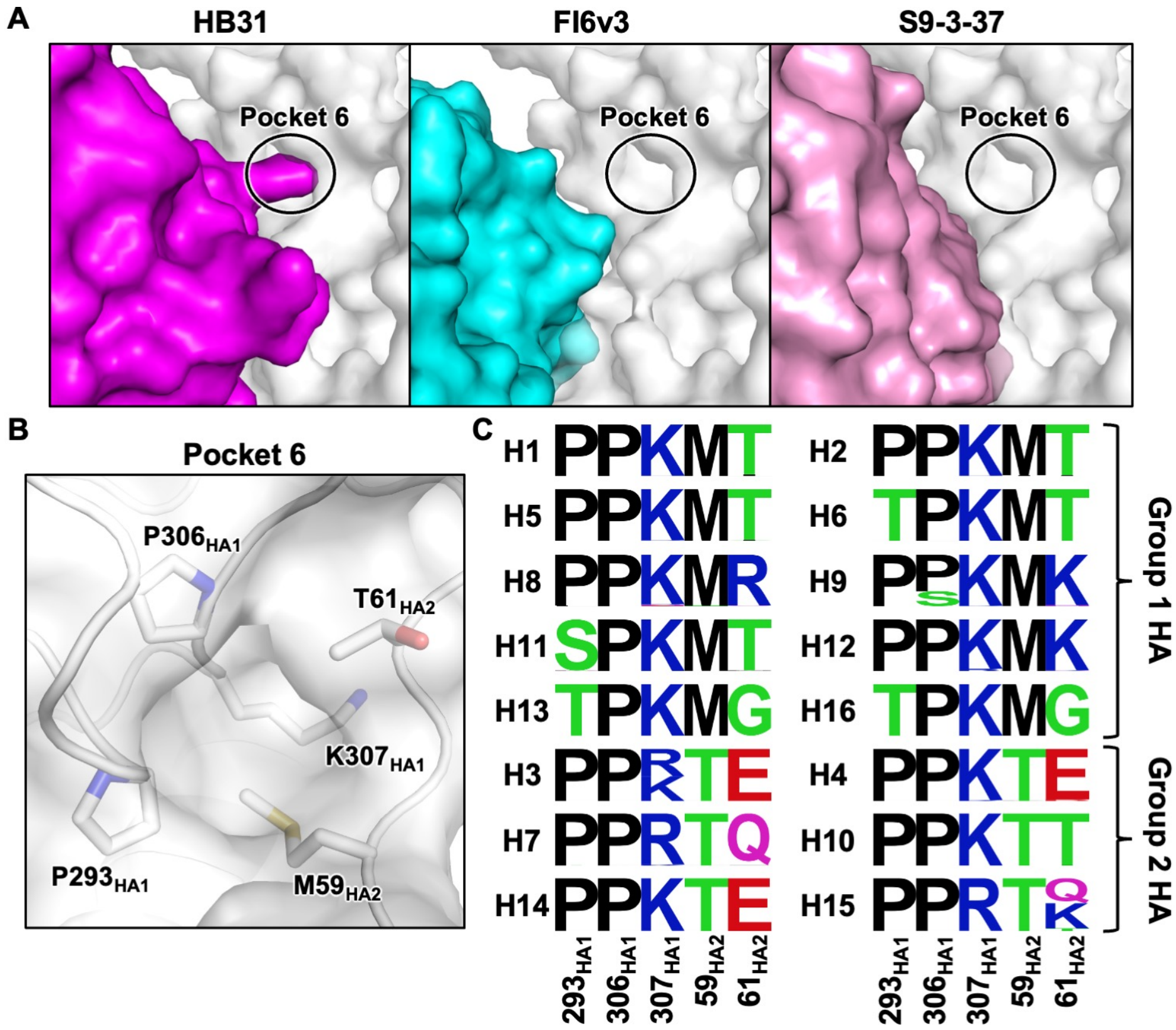

Figure S12. Structure comparison of *IGHD3-9* HA stem antibodies and sequence conservation of pocket 6. (A) *IGHD3-9* HA stem antibodies HB31 (magenta), FI6v3 (PDB 3ZTN, cyan)[5] and S9-3-37 (PDB 6E3H, pink)[9], as well as HA (white) are shown as surface representation. The location of pocket 6 is indicated. **(B)** HA residues with side chains found in pocket 6 are shown in stick representation. **(C)** Sequence conservation of 293_{HA1}, 306_{HA1}, 307_{HA1}, 59_{HA2}, and 61_{HA2} in the indicated HA subtype is shown as a sequence logo.

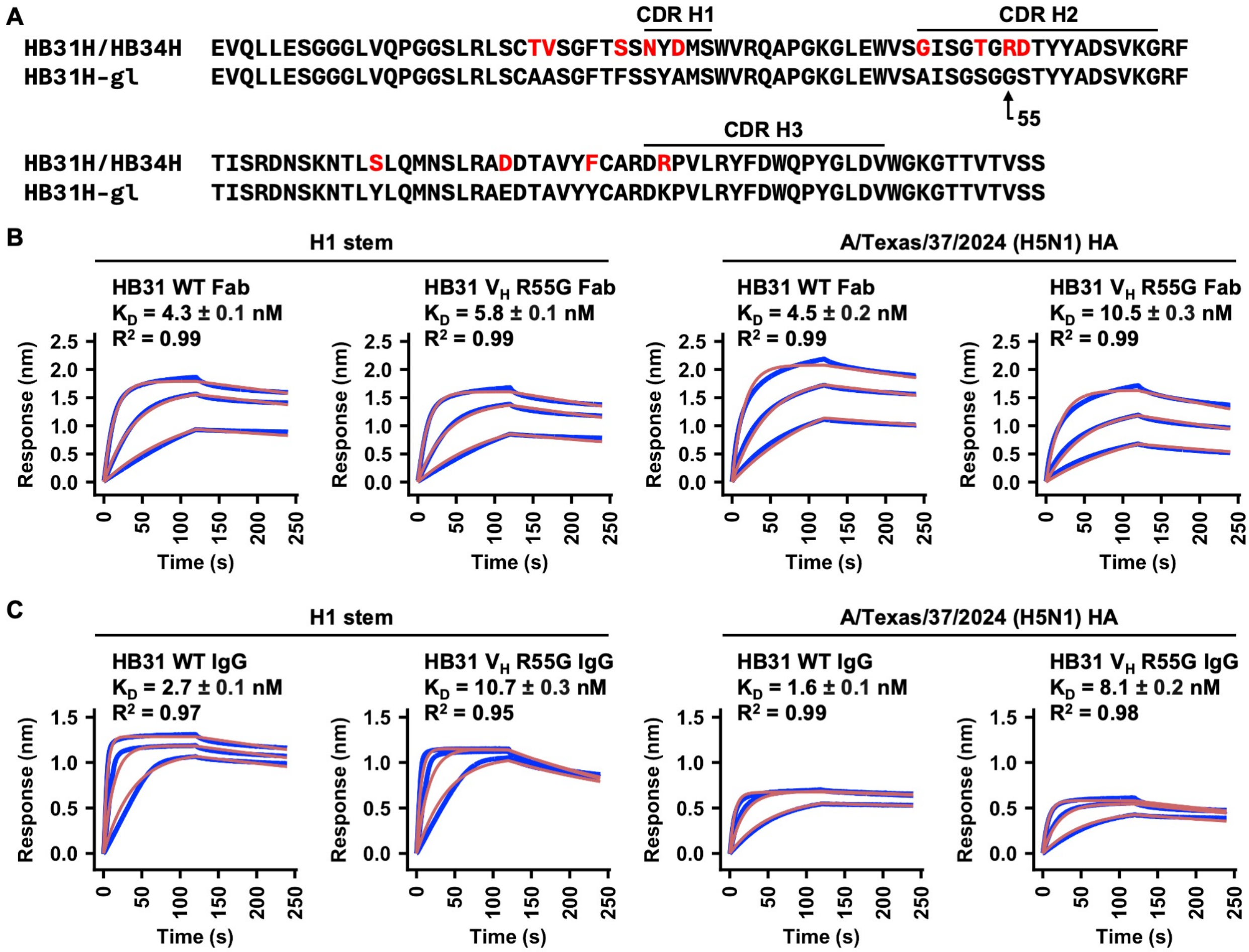

Figure S13. Sequence determinants for the upper HA stem pocket of HB31. (A) Sequence alignment of HB31 heavy-chain variable domain with its inferred germline sequence was performed using MAFFT[1]. Residues that represent somatic hypermutations in the V gene are colored in red. **(B-C)** Binding kinetics of HB31 wild-type (WT) and V_H R55G germline revertant **(B)** in Fab format or **(C)** IgG format against H1 stem and A/Texas/37/2024 (H5N1) HA were measured by biolayer interferometry (BLI). Y-axis represents the response. Blue and red lines represent the response curve and the 1:1 binding model, respectively. Binding kinetics were measured for 300 nM, 100 nM, and 33 nM of Fab. The dissociation constants (K_D) and the goodness-of-fit values (R^2) are shown. K_D values are shown as mean ± standard deviation.

SUPPLEMENTAL REFERENCES

1 Katoh, K. & Standley, D. M. MAFFT multiple sequence alignment software version 7: improvements in performance and usability. *Mol Biol Evol* **30**, 772-780 (2013). https://doi.org/10.1093/molbev/mst010

2 Dreyfus, C. *et al.* Highly conserved protective epitopes on influenza B viruses. *Science* **337**, 1343-1348 (2012). https://doi.org/10.1126/science.1222908

3 Lin, T. H. *et al.* Structurally convergent antibodies derived from different vaccine strategies target the influenza virus HA anchor epitope with a subset of V(H)3 and V(K)3 genes. *Nat Commun* **16**, 1268 (2025). https://doi.org/10.1038/s41467-025-56496-4

4 Guthmiller, J. J. *et al.* Broadly neutralizing antibodies target a haemagglutinin anchor epitope. *Nature* **602**, 314-320 (2022). https://doi.org/10.1038/s41586-021-04356-8

5 Corti, D. *et al.* A neutralizing antibody selected from plasma cells that binds to group 1 and group 2 influenza A hemagglutinins. *Science* **333**, 850-856 (2011). https://doi.org/10.1126/science.1205669

6 Kallewaard, N. L. *et al.* Structure and function analysis of an antibody recognizing all influenza A subtypes. *Cell* **166**, 596-608 (2016). https://doi.org/10.1016/j.cell.2016.05.073

7 Fu, Y. *et al.* A broadly neutralizing anti-influenza antibody reveals ongoing capacity of haemagglutinin-specific memory B cells to evolve. *Nat Commun* **7**, 12780 (2016). https://doi.org/10.1038/ncomms12780

8 Liu, X., Balligand, T., Le Gall, C. & Ploegh, H. L. A monoclonal anti-hemagglutinin stem antibody modified with zanamivir protects against both influenza A and B viruses. *Proc Natl Acad Sci U S A* **122**, e2424889122 (2025). https://doi.org/10.1073/pnas.2424889122

9 Wu, N. C. *et al.* Recurring and adaptable binding motifs in broadly neutralizing antibodies to influenza virus are encoded on the D3-9 segment of the Ig gene. *Cell Host Microbe* **24**, 569-578.e564 (2018). https://doi.org/10.1016/j.chom.2018.09.010
