# Supplementary material for: Structural basis for distinct protective mechanisms of IGHV3-23 antibodies targeting influenza hemagglutinin stem
Source: Nat Commun. 2026 Jun 22;17:7822. doi: 10.1038/s41467-026-74658-w (PMC13438699; doi:10.1038/s41467-026-74658-w)
Supplement: Supplementary file 2 [file 41467_2026_74658_MOESM2_ESM.pdf]

nature portfolio

Corresponding author(s): Nicholas Wu, Chris Mok

Last updated by author(s): 05/28/2026

Reporting Summary

Nature Portfolio wishes to improve the reproducibility of the work that we publish. This form provides structure for consistency and transparency in reporting. For further information on Nature Portfolio policies, see our Editorial Policies and the Editorial Policy Checklist.

Statistics

For all statistical analyses, confirm that the following items are present in the figure legend, table legend, main text, or Methods section.

n/a	Confirmed	
☐	☒	The exact sample size (*n*) for each experimental group/condition, given as a discrete number and unit of measurement
☐	☒	A statement on whether measurements were taken from distinct samples or whether the same sample was measured repeatedly
☐	☒	The statistical test(s) used AND whether they are one- or two-sided<br>*Only common tests should be described solely by name; describe more complex techniques in the Methods section.*
☒	☐	A description of all covariates tested
☒	☐	A description of any assumptions or corrections, such as tests of normality and adjustment for multiple comparisons
☐	☒	A full description of the statistical parameters including central tendency (e.g. means) or other basic estimates (e.g. regression coefficient) AND variation (e.g. standard deviation) or associated estimates of uncertainty (e.g. confidence intervals)
☐	☒	For null hypothesis testing, the test statistic (e.g. *F*, *t*, *r*) with confidence intervals, effect sizes, degrees of freedom and *P* value noted<br>*Give P values as exact values whenever suitable.*
☒	☐	For Bayesian analysis, information on the choice of priors and Markov chain Monte Carlo settings
☒	☐	For hierarchical and complex designs, identification of the appropriate level for tests and full reporting of outcomes
☒	☐	Estimates of effect sizes (e.g. Cohen's *d*, Pearson's *r*), indicating how they were calculated

Our web collection on statistics for biologists contains articles on many of the points above.

Software and code

Policy information about availability of computer code

Data collection	High-resolution data were acquired on a Titan Krios microscope equipped with a Gatan detector.
Data analysis	Single-particle cryo-EM data were processed using CryoSPARC Live (v4.5), and the density map was sharpened using DeepEMhancer (v0.15). The initial model for the cryo-EM maps was built using ModelAngelo (v0.3). This model was fitted into the cryo-EM density map using UCSF Chimera (v1.16), followed by manual adjustments in COOT (v0.9.8.1) and refinement with the PHENIX real-space refinement program (v1.20). Custom scripts for analyzing the PacBio sequencing data have been deposited to https://zenodo.org/records/20435345.

For manuscripts utilizing custom algorithms or software that are central to the research but not yet described in published literature, software must be made available to editors and reviewers. We strongly encourage code deposition in a community repository (e.g. GitHub). See the Nature Portfolio guidelines for submitting code & software for further information.

Data

Policy information about availability of data

All manuscripts must include a data availability statement. This statement should provide the following information, where applicable:

- Accession codes, unique identifiers, or web links for publicly available datasets
- A description of any restrictions on data availability
- For clinical datasets or third party data, please ensure that the statement adheres to our policy

PacBio sequencing data generated in this study have been submitted to the NIH Short Read Archive under accession number: BioProject PRJNA1104662 [https://www.ncbi.nlm.nih.gov/bioproject/PRJNA1104662]. Cryo-EM maps have been deposited to the Electron Microscopy Data Bank under accession codes: EMD-70809

[https://www.ebi.ac.uk/emdb/EMD-70809], EMD-70810 [https://www.ebi.ac.uk/emdb/EMD-70810], and EMD-70811 [https://www.ebi.ac.uk/emdb/EMD-70811]. The refined models have been deposited to the RCSB Protein Data Bank under accession codes 9OSS [https://doi.org/10.2210/pdb9OSS/pdb], 9OST [https://doi.org/10.2210/pdb9OST/pdb], and 9OSU [https://doi.org/10.2210/pdb9OSU/pdb]. Source Data are provided with this paper.

Research involving human participants, their data, or biological material

Policy information about studies with human participants or human data. See also policy information about sex, gender (identity/presentation), and sexual orientation and race, ethnicity and racism.

Reporting on sex and gender	*Use the terms sex (biological attribute) and gender (shaped by social and cultural circumstances) carefully in order to avoid confusing both terms. Indicate if findings apply to only one sex or gender; describe whether sex and gender were considered in study design; whether sex and/or gender was determined based on self-reporting or assigned and methods used. Provide in the source data disaggregated sex and gender data, where this information has been collected, and if consent has been obtained for sharing of individual-level data; provide overall numbers in this Reporting Summary. Please state if this information has not been collected. Report sex- and gender-based analyses where performed, justify reasons for lack of sex- and gender-based analysis.*
Reporting on race, ethnicity, or other socially relevant groupings	*Please specify the socially constructed or socially relevant categorization variable(s) used in your manuscript and explain why they were used. Please note that such variables should not be used as proxies for other socially constructed/relevant variables (for example, race or ethnicity should not be used as a proxy for socioeconomic status). Provide clear definitions of the relevant terms used, how they were provided (by the participants/respondents, the researchers, or third parties), and the method(s) used to classify people into the different categories (e.g. self-report, census or administrative data, social media data, etc.) Please provide details about how you controlled for confounding variables in your analyses.*
Population characteristics	*Describe the covariate-relevant population characteristics of the human research participants (e.g. age, genotypic information, past and current diagnosis and treatment categories). If you filled out the behavioural & social sciences study design questions and have nothing to add here, write "See above."*
Recruitment	*Describe how participants were recruited. Outline any potential self-selection bias or other biases that may be present and how these are likely to impact results.*
Ethics oversight	*Identify the organization(s) that approved the study protocol.*

Note that full information on the approval of the study protocol must also be provided in the manuscript.

Field-specific reporting

Please select the one below that is the best fit for your research. If you are not sure, read the appropriate sections before making your selection.

- [x] Life sciences
- [ ] Behavioural & social sciences
- [ ] Ecological, evolutionary & environmental sciences

For a reference copy of the document with all sections, see nature.com/documents/nr-reporting-summary-flat.pdf

Life sciences study design

All studies must disclose on these points even when the disclosure is negative.

Sample size	Experimental groups included 5 mice. Sample sizes were determined based on prior experience and established practice in influenza infection studies, where groups of 5 mice are enough to observe differences in weight loss and survival by minimizing animal use while ensuring statistical power.
Data exclusions	No data was excluded
Replication	All experiments were performed in duplicate to ensure reproducibility, and all attempts at replication were successful.
Randomization	All data obtained from mice in each experimental group were included in this study without randomization.
Blinding	*Describe whether the investigators were blinded to group allocation during data collection and/or analysis. If blinding was not possible, describe why OR explain why blinding was not relevant to your study.*

Reporting for specific materials, systems and methods

We require information from authors about some types of materials, experimental systems and methods used in many studies. Here, indicate whether each material, system or method listed is relevant to your study. If you are not sure if a list item applies to your research, read the appropriate section before selecting a response.

Materials & experimental systems

n/a	Involved in the study
☐	☒ Antibodies
☐	☒ Eukaryotic cell lines
☒	☐ Palaeontology and archaeology
☐	☒ Animals and other organisms
☒	☐ Clinical data
☒	☐ Dual use research of concern
☒	☐ Plants

Methods

n/a	Involved in the study
☒	☐ ChIP-seq
☒	☐ Flow cytometry
☒	☐ MRI-based neuroimaging

Antibodies

Antibodies used	For ELISA, an anti-human IgG secondary antibody (goat anti-human IgG, HRP-conjugated; Thermo Fisher Scientific, Cat. #31410) was used at a 1:5000 dilution. Custom antibodies (full IgG and Fab) were cloned into IgG1 phcmv backbones or mouse IgG2c and expressed in Expi293F cells (Thermo Fisher Scientific) following the manufacturer's protocol.
Validation	All commercial antibodies were validated by the manufacturer.

Eukaryotic cell lines

Policy information about cell lines and Sex and Gender in Research

Cell line source(s)	Human embryonic kidney 293T cells (ATCC,Cat. No. CRL-3216), MDCK-SIAT1 cells (Sigma-Aldrich, Cat. No. 05071502), Expi293F cells (Thermo Fisher Scientific, Cat. No. A14527) and Sf9 insect cells (ATCC, Cat. No. CRL-1711)were used in this study.
Authentication	These cells are routinely maintained in our lab. No other authentication at the lab level was performed.
Mycoplasma contamination	All cell lines were tested negative for mycoplasma contamination.
Commonly misidentified lines (See ICLAC register)	No commonly misidentified cell lines was used in this study.

Animals and other research organisms

Policy information about studies involving animals; ARRIVE guidelines recommended for reporting animal research, and Sex and Gender in Research

Laboratory animals	6-8 week old female BALB/c mice (Jackson Laboratory)
Wild animals	This study does not involve any wild animals.
Reporting on sex	Only female BALB/c mice were used in this study. Female mice were chosen because they are less aggressive and show reduced variability in humoral immune responses compared with male BALB/c mice. As the aim of the study was to evaluate the protective capacity of antibodies against the human virus, the sex of the animals was not expected to significantly influence the results or conclusions.
Field-collected samples	Mice were kept at 18-22 degrees with 40-60% humidity. Housing condition was 12h light/12h dark cycle.
Ethics oversight	The animal experiments were performed in accordance with protocols approved by UIUC Institutional Animal Care and Use Committee (IACUC), protocol number: 22215).

Note that full information on the approval of the study protocol must also be provided in the manuscript.

Plants

Seed stocks	*Report on the source of all seed stocks or other plant material used. If applicable, state the seed stock centre and catalogue number. If plant specimens were collected from the field, describe the collection location, date and sampling procedures.*
Novel plant genotypes	*Describe the methods by which all novel plant genotypes were produced. This includes those generated by transgenic approaches, gene editing, chemical/radiation-based mutagenesis and hybridization. For transgenic lines, describe the transformation method, the number of independent lines analyzed and the generation upon which experiments were performed. For gene-edited lines, describe the editor used, the endogenous sequence targeted for editing, the targeting guide RNA sequence (if applicable) and how the editor was applied.*
Authentication	*Describe any authentication procedures for each seed stock used or novel genotype generated. Describe any experiments used to assess the effect of a mutation and, where applicable, how potential secondary effects (e.g. second site T-DNA insertions, mosiacism, off-target gene editing) were examined.*
