# Supplementary material for: Structural basis for distinct protective mechanisms of IGHV3-23 antibodies targeting influenza hemagglutinin stem
Source: Nat Commun. 2026 Jun 22;17:7822. doi: 10.1038/s41467-026-74658-w (PMC13438699; doi:10.1038/s41467-026-74658-w)
Supplement: Supplementary file 3 [file 41467_2026_74658_MOESM3_ESM.pdf]

nature portfolio

Peer Review File

Structural basis for distinct protective mechanisms of IGHV3-23 antibodies targeting influenza hemagglutinin stem

Corresponding Author: Professor Nicholas Wu

This file contains all reviewer reports in order by version, followed by all author rebuttals in order by version.

Version 0:

Reviewer comments:

Reviewer #1

(Remarks to the Author)
Lv et al., identified three IGHV3-23-encoded influenza HA stem-targeting antibodies: HB31, HB34, and HB315 from phage-display human antibody libraries. All three antibodies showed varied level of binding activities across H1, H2 and H5 HAs. HB31 and HB34 displayed minimal neutralizing activity, but they conferred in vivo function which was comparable to, or even better than that of the potently neutralizing HB315. The protection by HB31 and HB34 could be largely due to their Fc-mediated effector functions. Cryo-EM structural analyses revealed that differences in epitope location and approach angles may be associated with distinct Fc accessibility and function observed among these antibodies.

The authors described biological and structural features of three cross-reactive anti-influenza A stem antibodies, two of which exhibited in vivo function primarily through the Fc-mediated effector function. Similar anti-stem human antibodies were reported earlier (Fu et al., Nat Commun 2016, Harshbarger et al., Nat Commun 2021, DiLillo et al., J Clin Invest 2016). The major concern is that HB31 and HB34 provided moderate protection against the VN1203 H5-PR8 in vivo, but both antibodies seem to have lost or minimal binding reactivity with more contemporary FL22, WA14 and Neth14 H5s.

A few minor comments are as follows,
1. Please include the results of RP8 HA protein in the binding activity (as shown in Figure 2B)
2. The epitopes of antibodies mapped above the aligned sequences could be helpful for explaining the binding breadth of antibodies across H1, H2 and H5 HAs.
3. In the Methods section, there was a part titled “Prophylactic and therapeutic protection experiments,” but in the manuscript (Figure 3), only the prophylactic results were shown. Should the therapeutic results have also been included?
4. Since the experimental results did not include therapeutic findings, it might be better to revise the Discussion section accordingly.

(Remarks on code availability)

Reviewer #2

(Remarks to the Author)
The manuscript by Lv and colleagues is a interesting study. It describes the detailed structure-function characterisation of three human antibodies against the influenza stem and makes a thorough comparison with the epitopes of existing stem binding antibodies. Their prophylactic efficacy in a mouse model was tested and how this relates to their structure-function is reported. This provides further understanding of the role that effector functions can play in viral neutralisation by stem binding antibodies. In addition it further highlights that antibodies that are non-neutralising in vitro tests may still have potency in vivo through effector functions. Another noteworthy result is that HB31/34 targets a pocket in the upper HA stem which is rarely targeted by other stem antibodies and that the orientation of the antibodies is suggested to facilitate better accessibility to Fcg receptors on effector cells so mediating ADCC.
The paper will be of general interest to those study mAbs in infectious disease and particularly those designing broad reactive vaccines and therapeutics in preparation for the next influenza pandemic. The paper is well written and the data supports the general conclusions although it could be improved in certain areas.
1. Data on the performance of HB31/34 vs HB315 in a therapeutic setting does not appear to be included in Figure 3.

Testing in a therapeutic setting is described in the methods section suggesting that this was done. Although the therapeutic setting is clearly a higher bar for the antibodies to reach its inclusion would strengthen the paper and may shed further light on the relative contributions and timing of ADCC vs viral neutralisation following infection.
2. Although acknowledged in the paper a limitation of the in vivo testing is that a exact species specificity match between the antibody Fc and the Fcg Receptors is not used. Using either antibodies with a mouse Fc or using transgenic mice with human FcgR would have overcome this limitation and add greater weight to the conclusions about the role of ADCC.
3. The competition data in Figure 2B is difficult to follow and needs clarification. HB34 is shown as competing with HB31 which would be inconsistent with structural data. This figure should be a 5 x 5 square with all three antibodies identified in this study plus the two controls CR9114, FISW84 all tested against each other. There is also no FISW84 as first antibody in competition experiment in Figure S2. The line on 198 needs some clarification as the data does not seem to show this as in Figure S2.
4. The affinity data to support the importance of somatic mutation at position 55 interaction with pocket 6 is not that convincing. The affinity reduction in reverting R55 back to germline is marginal and may be within the error of the BLI assay (Figure 5E for H5N1) and (Figure S8B for H1N1). Is there any other analysis that could be done to make a more compelling case for the importance of this mutation in interacting with this new ‘pocket 6’. By looking at just the off rates (koff) which are concentration independent, a greater difference might be observed. Maybe a greater difference could be seen by looking at full IgG rather than the Fabs ? The koff and kon rate should also ideally be reported in addition to KD.
Minor comments
1. Figure 1B - PR8 (H1N1) and Viet04 (H5N1) should be included in the ELISA as they are the strains used in the mouse study.
2. HB31 over HB34 have exactly the same HC but different LC. It is interesting that these antibodies like many other stem binding antibodies use only their heavy chain for recognition, did the authors note any differences in the properties of these antibodies worthy of comment. Does the LC have any somatic mutations ? If so do the authors think these were functionally selected in donors or were they artificially paired during library construction ?
1. Do the authors have any thoughts why HB34 came out of the CM selection and HB315 out of the hyperphage ? What was the rationale for trying different helper phage
2. Fig S5. Somatic mutation underline should be made clearer and IGHJ6 germline sequence should be red in Fig S5A
3. The authors should include a assay positive control on Fig S3 C and D for the LALA-PG Fc knockout antibodies in ADCC assay. FI6v3 is the only stem binding epitope included as a control for ADCC (Figure S3 A,B) but the epitope of this antibody is not discussed or included in Figure 4. The relative performance of HB31/34, HB315 against other stem antibodies for which epitope and approach angles are discussed (Figure 4) should be included (e.g CR9114, FISW84 and also MEDI8852 if available).

(Remarks on code availability)

Reviewer #3

(Remarks to the Author)
Lv et al. identified three HA stem-targeting antibodies by screening a phage-displayed human antibody library and systematically characterized their binding properties and structural basis. The authors revealed that the epitope of HB315 spans multiple classical stem epitopes, namely the central stem and anchor epitopes, while HB31 and HB34 target pocket 6, a rarely targeted site in the upper HA stem. Crucially, the in vivo protective activity of HB31 and HB34 primarily depends on Fc-mediated effector functions rather than direct neutralization. However, the overall novelty of this manuscript is insufficient, particularly against the backdrop of numerous reports on HA stem-directed antibodies against influenza viruses. The three antibodies reported in this work are far inferior to the reported elite antibodies in terms of both the breadth of reactivity and the strength of neutralizing activity, raising my doubts about its suitability for publication in a journal of the caliber of Nature Communications.
Major issues:
1. While the author mentions different protective mechanisms of IGHV2-23 lineage antibodies, the elucidation of these mechanisms remains superficial. The analysis of the Fc-mediated mechanism, in particular, remains merely speculative, significantly diminishing the research depth of this work. Additionally, the analysis of the complex structures is also superficial and the biochemical data presented are insufficient to support the core conclusions.
2. The second part of the results (stating that 'HB315, HB31, and HB34 are group 1 HA-specific broadly neutralizing antibodies') constitutes an overstatement. These three antibodies did not demonstrate broadly neutralizing potential against group 1 viruses. Furthermore, the number of group 1 virus HAs or strains tested was limited. In the description and analysis of binding and neutralizing activities, comprehensive quantitative descriptions are required. Additionally, thorough head-to-head comparisons with well-known control antibodies (and perhaps more control antibodies are needed) should be conducted, rather than relying solely on qualitative descriptions.
3. The animal experiment section is equally perplexing. The conclusion that human Fc plays an important role in protection in the animal model, purportedly demonstrated through mouse experiments, is unconvincing. Furthermore, the authors lack any description or explanation of this finding within the main text of the Results section, which raises doubts about the reliability of this conclusion.
4. Another serious issue with the animal experiment section is that while the authors state using 5 mice per group, with monitoring of body weight, survival rate, and lung viral load, the measurement of lung viral load requires sacrificing animals to collect lung tissues on day 3 post-infection. The authors fail to clarify the specific experimental arrangement for these mice. Based on the figures, it appears the authors used only 2-3 mice per group for lung viral load testing. This firstly violates standard experimental protocols and the minimum n-value requirement for statistical analysis. Furthermore, the reduced

number of remaining mice is insufficient for survival rate analysis. Consequently, in my view, this fundamentally compromises the validity of the entire animal study.
5. Regarding the structural analysis, when the authors determined the structures of the complexes of the three antibodies, they introduced additional antibodies for co-complex preparation and structure determination. However, they provide no justification for this inclusion, making the purpose of adding these other antibodies perplexing. The authors analyze that the binding angle of the antibodies is crucial for their Fc function, but this angular information is not adequately quantified. In fact, given the high resolution of the reconstructions achieved, the binding angles of all antibodies should have been precisely quantified. Moreover, considering the importance the authors place on the angle, a thorough comparison of the binding angles of these three antibodies with those of other well-known, reported antibodies should be performed. Simultaneously, evaluating the change in ADCC activity before and after HA binding for the antibodies would have further validated the authors' hypothesis. Otherwise, based on merely vague angle differences, it is difficult to establish a direct link to the differences in therapeutic performance of the antibodies.

Special issues:
1. Line 188 indicates that "most anchor antibodies, which also have an upward approaching angle, lack ADCC activity." However, Figure S3 demonstrates that HB315 (with an upward approaching angle) exhibits ADCC activity, which is lost upon Fc mutation. The authors need to conduct a more detailed structural analysis and provide supplementary explanations to clarify this anomaly.
2. It is advisable to convert Figure 2A into a sequential alignment format. This would more intuitively display the homology and differences in the heavy and light chain variable region sequences of HB31, HB34, and HB315.
3. To ensure consistency in the experimental design, supplementary binding activity data for PR8 should be included.
4. Figure 3K-M, Lack of statistical difference analysis"
5. The legend positions in different subfigures of Figure 3 are inconsistent. Please adjust them to appropriate positions within each subfigure to enhance readability.
6. Figure 4 does not emphasize key information. More details should be added to label the footprints on the same HA surface to visually demonstrate the spatial distribution differences of epitopes.
7. The authors demonstrated that all three antibodies bind to HA via their heavy chains, with HB31 and HB34 exhibiting identical binding modes. Furthermore, they postulated that HB31's additional engagement with pocket 6 may correlate with Arg55 (R55) in its heavy chain. This raises a critical question: does the heavy chain of HB34 antibody also feature Arg55 at position 55? This information is pivotal and warrants thorough investigation.
8. Line 211 mentions that the interaction between HB31 and pocket 6 enhances binding affinity, but the observed affinity did not significantly decrease after mutation. This evidence is insufficient; additional biochemical experiments should be conducted to support this conclusion.
9. LALA-mutated antibodies must undergo validation of their in vitro activity before animal experiments, including two aspects: viral specificity remains unchanged, while Fc receptor binding activity undergoes alterations.
10. In Figures 5A and 5B, specific amino acid names and numbers should be added next to the HA structure or residues to enhance structural traceability.
11. Line 227 suggests that "pocket 6 can serve as a new target." It would be beneficial to include a conservation analysis of pocket 6 across different Group 1 HA subtypes to verify its potential as a broad-spectrum target.
12. In Figure S3's "Antibody-dependent cellular cytotoxicity (ADCC) reporter assay," both positive and negative controls should be incorporated into subfigures C and D to confirm the specificity of the experimental system.
13. A scale bar should be added to Figure S7A to clarify the actual resolution scale of the image.
14. The flowchart for cryo-EM data processing of the structures of HB31, HB34, and HB315 Fabs in complex with HA is overly simplistic. Adding multi-angle views of the cryo-EM density map to Figure S7 would more comprehensively show the spatial characteristics of the complex structure.
15. Line 493 mentions, "Due to insufficient binding of FISW84 Fab, the HB31 complex was ultimately processed with C1 symmetry and FISW84 was not structurally modeled." A further analysis is needed on the potential reasons for the insufficient binding of FISW84, whether its binding could be enhanced by optimizing complex preparation conditions, and how this situation may impact the interpretation of the binding mode between HB31 and HA.

(Remarks on code availability)

Version 1:

Reviewer comments:

Reviewer #1

(Remarks to the Author)
The authors have well addressed the comments and revised the manuscript accordingly.

(Remarks on code availability)

Reviewer #2

(Remarks to the Author)

The authors have addressed my comments. Specifically, the mouse in vivo studies have been improved with the additional testing using a mouse Fc and additional evaluation of potency in a therapeutic setting. Further testing the importance of the R55 HC somatic mutation and its interaction with pocket 6 have also been included using full IgG. In my view the paper is now acceptable for publication.

(Remarks on code availability)

Reviewer #3

(Remarks to the Author)
The authors have satisfactorily addressed my previous comments, and the manuscript has been further improved. I recommend the manuscript for publication.

(Remarks on code availability)

Open Access This Peer Review File is licensed under a Creative Commons Attribution 4.0 International License, which permits use, sharing, adaptation, distribution and reproduction in any medium or format, as long as you give appropriate credit to the original author(s) and the source, provide a link to the Creative Commons license, and indicate if changes were made.
In cases where reviewers are anonymous, credit should be given to 'Anonymous Referee' and the source.
The images or other third party material in this Peer Review File are included in the article's Creative Commons license, unless indicated otherwise in a credit line to the material. If material is not included in the article's Creative Commons license and your intended use is not permitted by statutory regulation or exceeds the permitted use, you will need to obtain permission directly from the copyright holder.
To view a copy of this license, visit https://creativecommons.org/licenses/by/4.0/

Reviewers' Comments:

Reviewer #1 (Remarks to the Author):

Lv et al., identified three IGHV3-23-encoded influenza HA stem-targeting antibodies: HB31, HB34, and HB315 from phage-display human antibody libraries. All three antibodies showed varied level of binding activities across H1, H2 and H5 HAs. HB31 and HB34 displayed minimal neutralizing activity, but they conferred in vivo function which was comparable to, or even better than that of the potently neutralizing HB315. The protection by HB31 and HB34 could be largely due to their Fc-mediated effector functions. Cryo-EM structural analyses revealed that differences in epitope location and approach angles may be associated with distinct Fc accessibility and function observed among these antibodies.

The authors described biological and structural features of three cross-reactive anti-influenza A stem antibodies, two of which exhibited in vivo function primarily through the Fc-mediated effector function. Similar anti-stem human antibodies were reported earlier (Fu et al., Nat Commun 2016, Harshbarger et al., Nat Commun 2021, DiLillo et al., J Clin Invest 2016). The major concern is that HB31 and HB34 provided moderate protection against the VN1203 H5-PR8 in vivo, but both antibodies seem to have lost or minimal binding reactivity with more contemporary FL22, WA14 and Neth14 H5s.

Response: We appreciate the reviewer's thoughtful comment. In the revised manuscript, we acknowledge that previous studies have shown the importance of Fc-mediated effector functions for the *in vivo* protection activity of HA stem antibodies:

> Lines 176-177: "Previous studies have shown that some HA stem antibodies confer protection *in vivo* primarily through Fc-mediated effector functions[48-50]."

Note that while both Fu et al., Nat Commun (2016) and Harshbarger et al., Nat Commun (2021) characterized the HA stem bnAb 3I14, they did not directly show that the *in vivo* protection activity of 3I14 was primarily through the Fc-mediated effector function. As a result, these two studies were not cited here.

We also agree that the protection of HB31 and HB34 against VN1203 H5-PR8 was moderate *in vivo*. However, the novelty of our study is the structural comparison of antibodies that protect through fundamentally distinct mechanisms, namely Fc-effector-dominant versus neutralization-dominant. To our knowledge, no prior study has correlated *in vivo* protection and ADCC activity of HA central stem antibodies to their binding geometries. Our findings provide new insights into the structure-function relationship of HA central stem antibodies.

A few minor comments are as follows,
1. Please include the results of RP8 HA protein in the binding activity (as shown in Figure 2B)

Response: The binding data against PR8 HA has been added to Figure 2B of the revised manuscript.

2. The epitopes of antibodies mapped above the aligned sequences could be helpful for explaining the binding breadth of antibodies across H1, H2 and H5 HAs.

Response: Thank you for the suggestion. We have performed the suggested analysis, which does help explain the higher binding breadth of HB315 compared to HB31 and HB34. The results are presented in Figure S10D-F of the revised manuscript and described in the Results section:

> Lines 227-237: "Our binding data above showed that the binding activity of HB315 to H2N2 HA was at least 4-fold stronger than HB31 and HB34 (Figure 2B). This difference was likely due to how they engaged HA2 residue 45 (Figure S10D-G). Although most HA subtypes possess an Ile at HA2 residue 45, human H2N2 HA has a Phe at this residue, which is known to limit the cross-reactivity of HA stem antibodies[25,52]. HB31, HB34, and HB315 all interacted with $I45_{HA2}$ via their CDR H3. At the same time, the CDR H3s of HB31 and HB34, but not HB315, were stabilized by light chain interactions (Figure S10G). Therefore, the bulkier Phe45 residue in H2N2 HA could be accommodated by the less constrained CDR H3 of HB315, whereas the more rigid CDR H3s of HB31 and HB34 would result in steric hindrance. This structural analysis explained why HB315, but not HB31 or HB34, strongly cross-reacted with H2N2 HA."

3. In the Methods section, there was a part titled "Prophylactic and therapeutic protection experiments," but in the manuscript (Figure 3), only the prophylactic results were shown. Should the therapeutic results have also been included?

Response: We have performed the therapeutic experiment of the three antibodies against PR8 H1N1 virus. The results are presented in Figure 3D-F of the revised manuscript and described in the Results section:

> Lines 163-166: "Moreover, therapeutic administration of the three antibodies at 5 mg/kg conferred partial protection against PR8 infection, with survival rates of 60% (3/5), 80% (4/5), and 60% (3/5) in mice treated with HB31, HB34, and HB315, respectively (Figure 3D-F)."

4. Since the experimental results did not include therapeutic findings, it might be better to revise the Discussion section accordingly.

Response: Results from the therapeutic experiment are added to the revised manuscript (see response to comment #3 above).

Reviewer #2 (Remarks to the Author):

The manuscript by Lv and colleagues is a interesting study. It describes the detailed structure-function characterisation of three human antibodies against the influenza stem and makes a thorough comparison with the epitopes of existing stem binding antibodies. Their prophylactic efficacy in a mouse model was tested and how this relates to their structure-function is reported. This provides further understanding of the role that effector functions can play in viral neutralisation by stem binding antibodies. In addition it further highlights that antibodies that are non-neutralising in vitro tests may still have potency in vivo through effector functions. Another noteworthy result is that HB31/34 targets a pocket in the upper HA stem which is rarely targeted by other stem antibodies and that the orientation of the antibodies is suggested to facilitate better accessibility to Fcg receptors on effector cells so mediating ADCC.
The paper will be of general interest to those study mAbs in infectious disease and particularly those designing broad reactive vaccines and therapeutics in preparation for the next influenza pandemic. The paper is well written and the data supports the general conclusions although it could be improved in certain areas.

Response: Thank you for the positive comments.

1. Data on the performance of HB31/34 vs HB315 in a therapeutic setting does not appear to be included in Figure 3. Testing in a therapeutic setting is described in the methods section suggesting that this was done. Although the therapeutic setting is clearly a higher bar for the antibodies to reach its inclusion would strengthen the paper and may shed further light on the relative contributions and timing of ADCC vs viral neutralisation following infection.

Response: We have performed the therapeutic experiment of the three antibodies against PR8 H1N1 virus. The results are presented in Figure 3D-F of the revised manuscript and described in the Results section:

> Lines 163-166: "Moreover, therapeutic administration of the three antibodies at 5 mg/kg conferred partial protection against PR8 infection, with survival rates of 60% (3/5), 80% (4/5), and 60% (3/5) in mice treated with HB31, HB34, and HB315, respectively (Figure 3D-F)."

2. Although acknowledged in the paper a limitation of the in vivo testing is that a exact species specificity match between the antibody Fc and the Fcg Receptors is not used. Using either antibodies with a mouse Fc or using transgenic mice with human FcgR would have overcome this limitation and add greater weight to the conclusions about the role of ADCC.

Response: We have tested the protection efficacy of HB31, HB34, and HB315 with murine Fc against PR8 H1N1 virus infection. The results are presented in Figure 3M-O of the revised manuscript and described in the Results section:

> Lines 189-194: "Furthermore, HB31 and HB34 with murine Fc conferred complete protection against PR8 at 1 mg/kg (Figure 3M-N). By contrast, HB315 with murine Fc only partial protected against PR8 with a survival rate of 40% (2/5) (Figure 3M-N). HB31 and HB34 with murine Fc also reduced lung viral titers by approximately 10-fold compared to their human Fc counterparts ($p = 0.01$ and 0.04, respectively, two-tailed t-test), whereas no such difference was observed for HB315 ($p = 0.42$, two-tailed t-test) (Figure 3O)."

3. The competition data in Figure 2B is difficult to follow and needs clarification. HB34 is shown as competing with HB31 which would be inconsistent with structural data. This figure should be a 5 x 5 square with all three antibodies identified in this study plus the two controls CR9114, FISW84 all tested against each other. There is also no FISW84 as first antibody in competition experiment in Figure S2. The line on 198 needs some clarification as the data does not seem to show this as in Figure S2.

Response: We have updated the competition data in the revised manuscript (Figure 2D and Figure S3). The competition heatmap in Figure 2D is now presented as a 5 x 5 matrix. Accordingly, Figure S3 has been updated to include the 25 corresponding sensorgrams. The Results section has been revised as follows:

> Lines 142-152: "To probe the epitopes of HB31, HB34, and HB315, we performed a binding competition assay using biolayer interferometry. This binding competition assay also included 047-09 4F04 (known as 4F04 hereinafter)[27,28] and CR9114[12] as controls, which are prototypic antibodies to the anchor and central stem epitopes, respectively. HB31, HB34, and HB315 showed 90%-100% competition with CR9114 (Figure 2D and

> Figure S3). By contrast, they exhibited no competition with 4F04 when 4F04 was added first (Figure 2D and Figure S3). These results suggested that HB31, HB34, and HB315 targeted the central stem epitope, similar to CR9114. Although HB31, HB34, and HB315 competed strongly with 4F04 when added first (Figure 2D and Figure S3), this observation was likely due to steric hindrance blocking access to the 4F04 epitope, which is located closer to the biosensor surface. Consistently, CR9114 had no competition with 4F04 when 4F04 was added first, but showed strong competition when the order was reversed."

Due to the weak binding affinity of FISW84, we have replaced it with 047-09 4F04 in the competition assay for higher quality data. Note that the competition between HB31 and HB34 is consistent with the structural data, which revealed a complete overlap in their epitopes.

The sentence in line 198 of the initial submission is now removed to avoid confusion.

4. The affinity data to support the importance of somatic mutation at position 55 interaction with pocket 6 is not that convincing. The affinity reduction in reverting R55 back to germline is marginal and may be within the error of the BLI assay (Figure 5E for H5N1) and (Figure S8B for H1N1). Is there any other analysis that could be done to make a more compelling case for the importance of this mutation in interacting with this new 'pocket 6'. By looking at just the off rates (koff) which are concentration independent, a greater difference might be observed. Maybe a greater difference could be seen by looking at full IgG rather than the Fabs? The koff and kon rate should also ideally be reported in addition to KD.

Response: To further support the importance of somatic mutation at position 55 interaction with pocket 6, we have measured the binding kinetics using IgG as suggested. Additionally, we have also measured the neutralization activity of HB31 WT and V_H R55G germline revertant. These results are presented in Figure 5E-G and Figure S13C, and described in the Results section of the revised manuscript:

> Lines 256-265: "The same V_H R55G revertant in IgG format weakened the K_D against A/Texas/37/2024 (H5N1) HA and H1 stem by 5- and 4-fold, respectively (Figure 5E and Figure S13C). A similar observation was also made for the dissociation rate (k_{off}) (Figure 5F and Figure S13B-C). Consistently, reverting V_H R55 to V_H G55 in HB31 reduced the neutralization potency against A/Chile/1/1983 (H1N1) and A/Beijing/262/1995 (H1N1), with the minimum inhibitory concentration (MIC) increasing from 75 μg/mL to 100 μg/mL (Figure 5G). Furthermore, HB31 neutralized A/Michigan/45/2015 (H1N1) with an MIC of 75 μg/mL, but no neutralization activity was observed for the V_H R55G revertant (Figure 5G). These results showed that engagement of pocket 6 moderately strengthened the binding of HB31 to HA stem."

Minor comments

1. Figure 1B - PR8 (H1N1) and Viet04 (H5N1) should be included in the ELISA as they are the strains used in the mouse study.

Response: The binding data against PR8 HA and Viet04 HA are added to Figure 2B of the revised manuscript.

2. HB31 over HB34 have exactly the same HC but different LC. It is interesting that these antibodies like many other stem binding antibodies use only their heavy chain for recognition, did the authors note any differences in the properties of these antibodies worthy of comment. Does

the LC have any somatic mutations? If so do the authors think these were functionally selected in donors or were they artificially paired during library construction?

Response: We have identified the differences of the heavy-light chain interfaces between HB31 and HB34. Additionally, we have also measured the thermostability of HB31 and HB34. These results are included in Figure S9D-E and described in the Results section of the revised manuscript:

> Lines 205-212: "Since HB31 and HB34 shared the same heavy chain sequence, their binding modes were almost identical. Nevertheless, their light chain sequences differed, resulting in distinct heavy-light chain interfaces. A notable difference was tighter packing of V_L Y96 with the heavy chain in HB31 compared to HB34 (Figure S9D). In HB31, V_L Y96 H-bonded with V_H Y100g and was 7 Å from V_H L100i. In HB34, V_L Y96 H-bonded with V_H R96 and was only 3 Å from V_H L100i (Figure S9D). This difference is likely responsible for the higher thermostability of HB34 (T_m = 68°C) relative to HB31 (T_m = 66.5°C) (Figure S9E)."

While the light chains of HB31, HB34, and HB315 contain somatic hypermutations, we currently lack the data to conclude whether these antibodies were functionally selected in donors or are the result of artificial pairing.

1. Do the authors have any thoughts why HB34 came out of the CM selection and HB315 out of the hyperphage? What was the rationale for trying different helper phage

Response: Hyperphage displays more antibody copies per phage particle than does the CM phage. Consequently, hyperphage allows multivalent binding, which should in theory facilitate identification of weaker binders. Nevertheless, HB315, which came out from the hyperphage screen, has a better binding affinity than HB34. While the underlying cause for the lack of weaker binders in our hyperphage-based selection remains to be determined, we believe such an investigation is outside the primary scope of this manuscript.

2. Fig S5. Somatic mutation underline should be made clearer and IGHJ6 germline sequence should be red in Fig S5A

Response: Fixed. Note that Figure S5 in the initial submission is now Figure S11 in the revised manuscript.

3. The authors should include a assay positive control on Fig S3 C and D for the LALA-PG Fc knockout antibodies in ADCC assay. FI6v3 is the only stem binding epitope included as a control for ADCC (Figure S3 A,B) but the epitope of this antibody is not discussed or included in Figure 4. The relative performance of HB31/34, HB315 against other stem antibodies for which epitope and approach angles are discussed (Figure 4) should be included (e.g CR9114, FISW84 and also MEDI8852 if available).

Response: We have updated the Figure S5 (i.e. Figure S3 in the initial submission) to include three other stem antibodies CR9114, MEDI8852, and 047-09 4F04. We also included the epitope analysis for FI6v3 and 047-09 4F04 in Figure 4. Of note, we have replaced FISW84 with 047-09 4F04, which also targets the anchor epitope, throughout the manuscript (also see our response to major comment #3 above).

Reviewer #3 (Remarks to the Author):

Lv et al. identified three HA stem-targeting antibodies by screening a phage-displayed human antibody library and systematically characterized their binding properties and structural basis. The authors revealed that the epitope of HB315 spans multiple classical stem epitopes, namely the central stem and anchor epitopes, while HB31 and HB34 target pocket 6, a rarely targeted site in the upper HA stem. Crucially, the in vivo protective activity of HB31 and HB34 primarily depends on Fc-mediated effector functions rather than direct neutralization. However, the overall novelty of this manuscript is insufficient, particularly against the backdrop of numerous reports on HA stem-directed antibodies against influenza viruses. The three antibodies reported in this work are far inferior to the reported elite antibodies in terms of both the breadth of reactivity and the strength of neutralizing activity, raising my doubts about its suitability for publication in a journal of the caliber of Nature Communications.

Response: Thank you for the comments. We agree that the HA stem antibodies identified in our study were inferior to many that were previously reported. However, the major novelty of our study is the structural comparison of antibodies that protect through fundamentally distinct mechanisms, namely Fc-effector-dominant versus neutralization-dominant. To our knowledge, no prior study has correlated *in vivo* protection and ADCC activity of HA central stem antibodies to their binding geometries. Therefore, our findings provide new insights into the structure-function relationship of HA central stem antibodies, which have important implications for designing broad reactive vaccines and therapeutics, as also mentioned by reviewer #2.

Major issues:
1. While the author mentions different protective mechanisms of IGHV2-23 lineage antibodies, the elucidation of these mechanisms remains superficial. The analysis of the Fc-mediated mechanism, in particular, remains merely speculative, significantly diminishing the research depth of this work. Additionally, the analysis of the complex structures is also superficial and the biochemical data presented are insufficient to support the core conclusions.

Response: The following experiments and analyses are added to the revised manuscript to further support our core conclusions:

1. To substantiate the importance of Fc-mediated effector functions in the *in vivo* efficacy of HB31 and HB34, we added a prophylactic protection experiment by replacing the human Fc of the three antibodies with murine Fc. The results are shown in Figure 3M-O of the revised manuscript and described in the Results section:

   Lines 189-194: "Consistently, Furthermore, HB31 and HB34 with murine Fc conferred complete protection against PR8 at 1 mg/kg (Figure 3M-N). By contrast, HB315 with murine Fc only partial protected against PR8 with a survival rate of 40% (2/5) (Figure 3M-N). HB31 and HB34 with murine Fc also reduced lung viral titers by approximately 10-fold compared to their human Fc counterparts ($p = 0.01$ and 0.04, respectively, two-tailed t-test), whereas no such difference was observed for HB315 ($p = 0.42$, two-tailed t-test) (Figure 3O)."

2. To provide a better mechanistic understanding of the functional differences between HB31, and HB34, which shared the same heavy chain sequences but with different light chain sequences, we added a more detailed analysis of the V_H-V_L interface and measured the thermostability of HB31 and HB34. The results are shown in Figure S9D-E and described in the Results section of the revised manuscript:

Lines 205-212: “Since HB31 and HB34 shared the same heavy chain sequence, their binding modes were almost identical. Nevertheless, their light chain sequences differed, resulting in distinct heavy-light chain interfaces. A notable difference was tighter packing of V_L Y96 with the heavy chain in HB31 compared to HB34 (Figure S9D). In HB31, V_L Y96 H-bonded with V_H Y100g and was 7 Å from V_H L100i. In HB34, V_L Y96 H-bonded with V_H R96 and was only 3 Å from V_H L100i (Figure S9D). This difference is likely responsible for the higher thermostability of HB34 (T_m = 68°C) relative to HB31 (T_m = 66.5°C) (Figure S9E).”

3. The importance of somatic hypermutation V_H G55R mutation and its interaction with pocket 6 was further supported by the addition of a biolayer interferometry experiment with antibodies in IgG format as well as a neutralization assay. The results are shown in Figure 5E-G and Figure S13C, and described in the Results section of the revised manuscript:

   Lines 256-265: “The same V_H R55G revertant in IgG format weakened the K_D against A/Texas/37/2024 (H5N1) HA and H1 stem by 5- and 4-fold, respectively (Figure 5E and Figure S13C). A similar observation was also made for the dissociation rate (k_{off}) (Figure 5F and Figure S13B-C). Consistently, reverting V_H R55 to V_H G55 in HB31 reduced the neutralization potency against A/Chile/1/1983 (H1N1) and A/Beijing/262/1995 (H1N1), with the minimum inhibitory concentration (MIC) increasing from 75 μg/mL to 100 μg/mL (Figure 5G). Furthermore, HB31 neutralized A/Michigan/45/2015 (H1N1) with an MIC of 75 μg/mL, but no neutralization activity was observed for the V_H R55G revertant (Figure 5G). These results showed that engagement of pocket 6 moderately strengthened the binding of HB31 to HA stem.”

4. To quantify the structural differences among HB31, HB34, HB315, and other HA stem antibodies, we added an analysis to measure the epitope location and approaching angles of these antibodies. The results are shown in Figure 4F and described in the Results section of the revised manuscript:

   Lines 212-217: “HB31 and HB34 bound at least 4 Å higher on the HA stem than canonical central stem antibodies, such as CR9114[12], FI6v3[14], and MEDI8852[13] (Figure 4F). In contrast, HB315 bound lower than canonical central stem antibodies. Compared to HB31 and HB34, HB315 bound 17 Å lower on the HA stem, yet remained above the lower stem antibody CR8020 and the anchor antibody 4F04. HB315 also exhibits a more downward binding angle (112°) than HB31 and HB34 (101°) (Figure 4F).”

2. The second part of the results (stating that 'HB315, HB31, and HB34 are group 1 HA-specific broadly neutralizing antibodies') constitutes an overstatement. These three antibodies did not demonstrate broadly neutralizing potential against group 1 viruses. Furthermore, the number of group 1 virus HAs or strains tested was limited. In the description and analysis of binding and neutralizing activities, comprehensive quantitative descriptions are required. Additionally, thorough head-to-head comparisons with well-known control antibodies (and perhaps more control antibodies are needed) should be conducted, rather than relying solely on qualitative descriptions.

Response: The subtitle for the second part of the results is revised to: “HB31, HB34, and HB315 are group 1 HA-specific antibodies”. The phrase “broadly neutralizing” is removed from the subtitle. The description for the binding and neutralizing activities in this result subsection is also revised to be more quantitative:

> Lines 117-122: “Nevertheless, enzyme-linked immunosorbent assay (ELISA) showed that HB31 and HB34 exhibited 2- to 4-fold weaker binding activity than HB315 against HAs from post-2009 pandemic H1N1 strains, including A/California/04/2009 (H1N1), A/Michigan/45/2015 (H1N1), and A/Brisbane/02/2018 (H1N1) (Figure 2B). Similarly, the binding activity of HB31 and HB34 to HAs from H2, H5, H6, and H9 subtypes was at least 2-fold weaker than HB315 (Figure 2B).”

> Lines 135-138: “Conversely, HB31 and HB34 were at least 50-fold less potent than HB315 against A/Chile/1/83 (H1N1), A/Beijing/262/95 (H1N1), and A/Brisbane/59/2007 (H1N1), and exhibited no detectable activity against the remaining strains, even at 100 μg/mL (Figure 2C).”

Note that we have added HAs from H6 and H9 subtypes to our binding analysis.

While our focus is on the comparison between HB31/HB34 and HB315, our revised manuscript has provided a quantitative description of the comparison with the control antibody FI6v3 in our binding and neutralization assays:

> Lines 122-126: “Despite the better binding activity and breadth of HB315, its binding activity to HAs from certain H5 strains, including A/northern pintail/WA/40964/2014 (H5N8), A/chicken/Netherlands/14015531/2014 (H5N8), as well as HAs from the H6 and H9 subtype was at least 2-fold weaker than the prototypic cross-group HA stem antibody FI6v3[14] (Figure 2B).”

> Lines 134-135: “The neutralization potency of HB315 was comparable to, or up to 4-fold greater than, that of FI6v3 against the tested viruses (Figure 2C).”

3. The animal experiment section is equally perplexing. The conclusion that human Fc plays an important role in protection in the animal model, purportedly demonstrated through mouse experiments, is unconvincing. Furthermore, the authors lack any description or explanation of this finding within the main text of the Results section, which raises doubts about the reliability of this conclusion.

Response: Our intention was not to claim that the effector function of human Fc is directly recapitulated in mice, but rather to test whether Fc-dependent mechanisms contribute to protection *in vivo*. In the revised manuscript, we have included additional experiments to substantiate the importance of Fc-effector functions in the *in vivo* efficacy of HB31 and HB34, by replacing their human Fc with a murine Fc. The results are presented in Figure 3M-O and described in the Results section of the revised manuscript:

> Lines 189-194: “Furthermore, HB31 and HB34 with murine Fc conferred complete protection against PR8 at 1 mg/kg (Figure 3M-N). By contrast, HB315 with murine Fc only partial protected against PR8 with a survival rate of 40% (2/5) (Figure 3M-N). HB31 and HB34 with murine Fc also reduced lung viral titers by approximately 10-fold compared to their human Fc counterparts ($p = 0.01$ and 0.04, respectively, two-tailed t-test), whereas no such difference was observed for HB315 ($p = 0.42$, two-tailed t-test) (Figure 3O).”

These new results provided additional data to substantiate our conclusion as stated in lines 187-188: “These results suggested that Fc effector functions were more important for the in vivo protection activity of HB31 and HB34 than for that of HB315.”

4. Another serious issue with the animal experiment section is that while the authors state using 5 mice per group, with monitoring of body weight, survival rate, and lung viral load, the measurement of lung viral load requires sacrificing animals to collect lung tissues on day 3 post-infection. The authors fail to clarify the specific experimental arrangement for these mice. Based on the figures, it appears the authors used only 2-3 mice per group for lung viral load testing. This firstly violates standard experimental protocols and the minimum n-value requirement for statistical analysis. Furthermore, the reduced number of remaining mice is insufficient for survival rate analysis. Consequently, in my view, this fundamentally compromises the validity of the entire animal study.

Response: We apologize for the lack of clarity regarding the experimental design of our mouse experiments in the initial submission, leading to this misunderstanding. Rather than having 5 mice per experimental group, a total of 8 mice were used per experimental group, with 5/8 for survival and weight monitoring, and 3/8 for lung viral titer measurement. We have made this clarification in the revised manuscript:

> Methods section (lines 510-515): "Per experimental group, a total of 8 female BALB/c mice at 6 weeks old were anesthetized with isoflurane and intranasally infected with 5× median lethal dose (LD50) of PR8 H1N1 virus or PR8-H5N1 (7:1 on backbone from A/Puerto Rico/8/1934). Mice were given the indicated antibody at a dose of 5 mg kg-1 intraperitoneally at 4 hours before infection (prophylaxis) or 24 hours after infection (therapeutics). Weight loss was monitored daily for 14 days in 5 out of 8 mice per experimental group."
>
> Figure 3 legend (lines 870-871): "A total of 8 mice were used per experimental group (n = 5 for survival and weight monitoring; n = 3 for measuring lung viral titers)."

5. Regarding the structural analysis, when the authors determined the structures of the complexes of the three antibodies, they introduced additional antibodies for co-complex preparation and structure determination. However, they provide no justification for this inclusion, making the purpose of adding these other antibodies perplexing. The authors analyze that the binding angle of the antibodies is crucial for their Fc function, but this angular information is not adequately quantified. In fact, given the high resolution of the reconstructions achieved, the binding angles of all antibodies should have been precisely quantified. Moreover, considering the importance the authors place on the angle, a thorough comparison of the binding angles of these three antibodies with those of other well-known, reported antibodies should be performed. Simultaneously, evaluating the change in ADCC activity before and after HA binding for the antibodies would have further validated the authors' hypothesis. Otherwise, based on merely vague angle differences, it is difficult to establish a direct link to the differences in therapeutic performance of the antibodies.

Response: In preliminary reconstructions using single Fab-HA complexes, preferred particle orientation and local flexibility of the stem region limited map quality. Inclusion of a non-competing Fab improved angular sampling and reconstruction stability without altering the binding interface of the antibody of interest. The rationale of including additional antibodies for co-complex preparation and structure determination is added to the Methods section of the revised manuscript:

> Lines 548-551: "FISW84 Fab was utilized exclusively as a tool to address the preferred orientation issues encountered during cryo-EM grid preparation of the HA-HB31 complex. Its addition to the complex potentially changed the physical and electrostatic properties of the complex, thereby facilitating more diverse particle orientations in the ice."

We have also performed a quantitative analysis of epitope location and antibody approaching angle. The results are presented in Figure 4F and described in the Results section of the revised manuscript.

> Lines 212-217: “HB31 and HB34 bound at least 4 Å higher on the HA stem than canonical central stem antibodies, such as CR9114[12], FI6v3[14], and MEDI8852[13] (Figure 4F). In contrast, HB315 bound lower than canonical central stem antibodies. Compared to HB31 and HB34, HB315 bound 17 Å lower on the HA stem, yet remained above the lower stem antibody CR8020 and the anchor antibody 4F04. HB315 also exhibits a more downward binding angle (112°) than HB31 and HB34 (101°) (Figure 4F).”

Special issues:

1. Line 188 indicates that "most anchor antibodies, which also have an upward approaching angle, lack ADCC activity." However, Figure S3 demonstrates that HB315 (with an upward approaching angle) exhibits ADCC activity, which is lost upon Fc mutation. The authors need to conduct a more detailed structural analysis and provide supplementary explanations to clarify this anomaly.

Response: We apologize for the confusion about HB315. HB315 targeted the central stem epitope but not the anchor epitope. In other words, although HB315 had an upward. approaching angle, its footprint has minimal overlap with that of the anchor antibody 4F04 (see Figure 4E of the revised manuscript). Clarification is also made in the Results section of the revised manuscript:

> Lines 203-204: “Consistent with the competition assay results (Figure 2D and Figure S3), our structural analysis showed that all three antibodies interacted with the central stem epitope (Figure 4A-E)…”

2. It is advisable to convert Figure 2A into a sequential alignment format. This would more intuitively display the homology and differences in the heavy and light chain variable region sequences of HB31, HB34, and HB315.

Response: The suggested sequence alignment is included in Figure S2 of the revised manuscript.

3. To ensure consistency in the experimental design, supplementary binding activity data for PR8 should be included.

Response: The binding data against PR8 HA is added to Figure 2B of the revised manuscript.

4. Figure 3K-M, Lack of statistical difference analysis"

Response: Statistic analysis is added to Figure 3C, F, I, L, and O of the revised manuscript.

5. The legend positions in different subfigures of Figure 3 are inconsistent. Please adjust them to appropriate positions within each subfigure to enhance readability.

Response: Fixed.

6. Figure 4 does not emphasize key information. More details should be added to label the footprints on the same HA surface to visually demonstrate the spatial distribution differences of epitopes.

Response: The highly conserved $W21_{HA2}$ is indicated in Figure 4E to visually demonstrate the spatial distribution differences of epitopes.

7. The authors demonstrated that all three antibodies bind to HA via their heavy chains, with HB31 and HB34 exhibiting identical binding modes. Furthermore, they postulated that HB31's additional engagement with pocket 6 may correlate with Arg55 (R55) in its heavy chain. This raises a critical question: does the heavy chain of HB34 antibody also feature Arg55 at position 55? This information is pivotal and warrants thorough investigation.

Response: As shown in the newly added sequence alignment (Figure S2A), HB31 and HB34 have the exact same heavy chain sequence. Thus, HB34 also has R55 in its heavy chain. This is clarified in the results section of the revised manuscript:

> Lines 251-253: "These structural observations for HB31 also applied to HB34, which shared the same heavy chain sequence as HB31 (Figure S2A) and displayed a binding mode almost identical to that of HB31 (Figure 4)."

8. Line 211 mentions that the interaction between HB31 and pocket 6 enhances binding affinity, but the observed affinity did not significantly decrease after mutation. This evidence is insufficient; additional biochemical experiments should be conducted to support this conclusion.

Response: The importance of somatic hypermutation V_H G55R mutation and its interaction with pocket 6 was further supported by the addition of a biolayer interferometry experiment with antibodies in IgG format as well as a neutralization assay. The results are shown in Figure 5E-G and Figure S13C, and described in the Results section of the revised manuscript:

> Lines 256-265: "The same V_H R55G revertant in IgG format weakened the K_D against A/Texas/37/2024 (H5N1) HA and H1 stem by 5- and 4-fold, respectively (Figure 5E and Figure S13C). A similar observation was also made for the dissociation rate (k_{off}) (Figure 5F and Figure S13B-C). Consistently, reverting V_H R55 to V_H G55 in HB31 reduced the neutralization potency against A/Chile/1/1983 (H1N1) and A/Beijing/262/1995 (H1N1), with the minimum inhibitory concentration (MIC) increasing from 75 µg/mL to 100 µg/mL (Figure 5G). Furthermore, HB31 neutralized A/Michigan/45/2015 (H1N1) with an MIC of 75 µg/mL, but no neutralization activity was observed for the V_H R55G revertant (Figure 5G). These results showed that engagement of pocket 6 moderately strengthened the binding of HB31 to HA stem."

9. LALA-mutated antibodies must undergo validation of their in vitro activity before animal experiments, including two aspects: viral specificity remains unchanged, while Fc receptor binding activity undergoes alterations.

Response: The binding activity and neutralization potency of the LALA-PG antibodies are now included in Figure S5C-D. The results are described in the Results section of the revised manuscript:

> Lines 179-181: "Notably, the binding and neutralization activities of the LALA-PG variants were comparable to their parental antibodies (Figure S5C-D)."

10. In Figures 5A and 5B, specific amino acid names and numbers should be added next to the HA structure or residues to enhance structural traceability.

Response: Added.

11. Line 227 suggests that "pocket 6 can serve as a new target." It would be beneficial to include a conservation analysis of pocket 6 across different Group 1 HA subtypes to verify its potential as a broad-spectrum target.

Response: The sequence conservation of pocket 6 is analyzed across different HA subtypes as suggested. The results are presented in Figure S12B-C of the revised manuscript and described in the Results section:

> Lines 265-267: "Notably, pocket 6, which consists of the side chains of $P293_{HA1}$, $P306_{HA1}$, $K307_{HA1}$, $M59_{HA2}$, and $T61_{HA2}$ (Figure S12B), is highly conserved among H1, H2, and H5 subtypes (Figure S12C)."

12. In Figure S3's "Antibody-dependent cellular cytotoxicity (ADCC) reporter assay," both positive and negative controls should be incorporated into subfigures C and D to confirm the specificity of the experimental system.

Response: Added. Please also see the legend of Figure S5:

> "FI6v3, CR9114 and MEDI8852 have previously been shown to exhibit ADCC activity[7,8], and therefore served as positive controls. IgG from human serum served as a negative control (IgG control)."

Note that Figure S3C-D in the initial submission is now Figure S5A-B in the revised manuscript.

13. A scale bar should be added to Figure S7A to clarify the actual resolution scale of the image.

Response: Added. Please see Figure S6A, S7A, and S8A of the revised manuscript.

14. The flowchart for cryo-EM data processing of the structures of HB31, HB34, and HB315 Fabs in complex with HA is overly simplistic. Adding multi-angle views of the cryo-EM density map to Figure S7 would more comprehensively show the spatial characteristics of the complex structure.

Response: Added. Please see Figure S6, S7, and S8 of the revised manuscript.

15. Line 493 mentions, "Due to insufficient binding of FISW84 Fab, the HB31 complex was ultimately processed with C1 symmetry and FISW84 was not structurally modeled." A further analysis is needed on the potential reasons for the insufficient binding of FISW84, whether its binding could be enhanced by optimizing complex preparation conditions, and how this situation may impact the interpretation of the binding mode between HB31 and HA.

Response: We appreciate the reviewer's attention to the technical details of the complex assembly. This is now clarified in the Methods section of the revised manuscript:

> Lines 545-557: "Due to substoichiometric quantities of bound FISW84 Fab in the HB31 complex, which was likely attributed to the weak binding affinity of FISW84 Fab, the data

were processed in C1 symmetry, and FISW84 Fab was not built into the map. FISW84 Fab was utilized exclusively as a tool to address the preferred orientation issues encountered during cryo-EM grid preparation of the HA-HB31 complex. Its addition to the complex potentially changed the physical and electrostatic properties of the complex, thereby facilitating more diverse particle orientations in the ice. Because FISW84 is a well-characterized antibody with a previously published structure (PDB ID: 6HJP)[54], and since it binds to a distinct anchor epitope that does not overlap with the HB31 stem epitope, its partial occupancy did not interfere with the high-resolution modeling of the HA-HB31 interface. The density for the HA trimer and the HB31 Fabs of interest remained clear and robust. As FISW84 was not the subject of our investigation, further optimization of its binding kinetics was deemed outside the scope of this structural study."

There are no further reviewers' comments that need to be addressed.
